# Eficácia de Cardiodesfibrilador Implantável na Prevenção Primária em Pacientes com Insuficiência Cardíaca com Fração de Ejeção Reduzida não Isquêmica

**DOI:** 10.36660/abc.20250675

**Published:** 2026-10-01

**Authors:** Luis E. Rohde, Leandro I. Zimerman, Lídia Z. Moura, Luis C. Danzmann, Silvia M. Martins, Verônica Colpani, Kelly Zucatti, José Albuquerque de Figueiredo-Neto, Fabiana G. Marcondes-Braga, Ricardo Mourilhe-Rocha, Denilson Campos de Albuquerque, Mucio T. de Oliveira-Júnior, Marcus V. Simões

**Affiliations:** 1 Hospital de Clínicas de Porto Alegre Porto Alegre RS Brasil Hospital de Clínicas de Porto Alegre, Porto Alegre, RS – Brasil; 2 Hospital Moinhos de Vento Porto Alegre RS Brasil Hospital Moinhos de Vento, Porto Alegre, RS – Brasil; 3 Hospital Universitário do Cajuru Pontifícia Universidade Católica Paraná Curitiba PR Brasil Hospital Universitário do Cajuru, Pontifícia Universidade Católica Paraná (PUCPR), Curitiba, PR – Brasil; 4 Santa Casa de Porto Alegre Porto Alegre RS Brasil Santa Casa de Porto Alegre, Porto Alegre, RS – Brasil; 5 Universidade Luterana do Brasil Canoas RS Brasil Universidade Luterana do Brasil, Canoas, RS – Brasil; 6 PROCAPE Universidade de Pernambuco Recife PE Brasil PROCAPE- Universidade de Pernambuco, Recife, PE – Brasil; 7 Hospital Sírio-Libanes São Paulo SP Brasil Hospital Sírio-Libanes, São Paulo, SP – Brasil; 8 Instituto de Cardiologia Fundação Universitária de Cardiologia Porto Alegre RS Brasil Instituto de Cardiologia/ Fundação Universitária de Cardiologia, Porto Alegre, RS – Brasil; 9 Hospital Universitário Universidade Federal do Maranhão São Luiz MA Brasil Hospital Universitário da Universidade Federal do Maranhão, São Luiz, MA – Brasil; 10 Instituto do Coração Hospital das Clínicas da Faculdade de Medicina Universidade de São Paulo São Paulo SP Brasil Instituto do Coração (InCor), Hospital das Clínicas da Faculdade de Medicina da Universidade de São Paulo, São Paulo, SP – Brasil; 11 Universidade do Estado do Rio de Janeiro Rio de Janeiro RJ Brasil Universidade do Estado do Rio de Janeiro, Rio de Janeiro, RJ – Brasil; 12 Complexo Hospitalar Américas - Vitória e Samaritano Barra Rio de Janeiro RJ Brasil Complexo Hospitalar Américas - Vitória e Samaritano Barra, Rio de Janeiro, RJ – Brasil; 13 Instituto D’Or de Pesquisa e Ensino Rio de Janeiro RJ Brasil Instituto D’Or de Pesquisa e Ensino-IDOR, Rio de Janeiro, RJ – Brasil; 14 Hospital das Clínicas Faculdade de Medicina de Ribeirão Preto Universidade de São Paulo Ribeirão Preto SP Brasil Hospital das Clínicas da Faculdade de Medicina de Ribeirão Preto da Universidade de São Paulo, Ribeirão Preto, SP – Brasil

**Keywords:** Insuficiência Cardíaca, Desfibriladores Implantáveis, Mortalidade

## Abstract

**Fundamento:**

A prevenção primária com cardiodesfibrilador implantável (CDI) constitui uma estratégia cujo papel vem sendo reavaliado na insuficiência cardíaca com fração de ejeção reduzida (ICFEr) não isquêmica.

**Objetivo:**

Sumarizar as evidências científicas disponíveis sobre o efeito do CDI na prevenção primária em pacientes com ICFEr não isquêmica.

**Métodos:**

Foi conduzida uma revisão sistemática rápida com metanálise, seguindo as recomendações do Cochrane Rapid Reviews Methods Group e do *checklist* Preferred Reporting Items for Systematic Reviews and Meta-Analyses. O nível de significância de 5% foi adotado. Buscas foram realizadas nas bases de dados MEDLINE, Embase e CENTRAL. Foram incluídos ensaios clínicos randomizados (ECRs) que avaliaram o implante de CDI para prevenção primária, com foco em morte súbita cardíaca (MSC), mortalidade cardiovascular e mortalidade por todas as causas.

**Resultados:**

Foram incluídas 18 publicações referentes a seis ECRs principais. Observou-se redução estatisticamente significativa da MSC (risco relativo [RR] 0,41; intervalo de confiança de 95% [IC95%] 0,26-0,65; alta certeza da evidência) e da mortalidade cardiovascular (RR 0,75; IC95% 0,59-0,95; alta certeza da evidência) nos grupos que receberam CDI. Em relação à mortalidade por todas as causas, a análise principal não demonstrou diferença estatisticamente significativa (RR 0,88; IC95% 0,72-1,07; moderada certeza da evidência). Na análise de subgrupo, estudos com menor tempo de seguimento (< 6,8 anos) apresentaram redução da mortalidade por todas as causas (RR 0,69; IC95% 0,52-0,92).

**Conclusão:**

Em pacientes com ICFEr não isquêmica, o CDI reduziu a MSC e a mortalidade cardiovascular, mas não houve redução da mortalidade por todas as causas na análise principal. As evidências apoiam seu uso, desde que fundamentado em avaliação clínica individualizada.

## Graphical abstract

**Figura Central:**
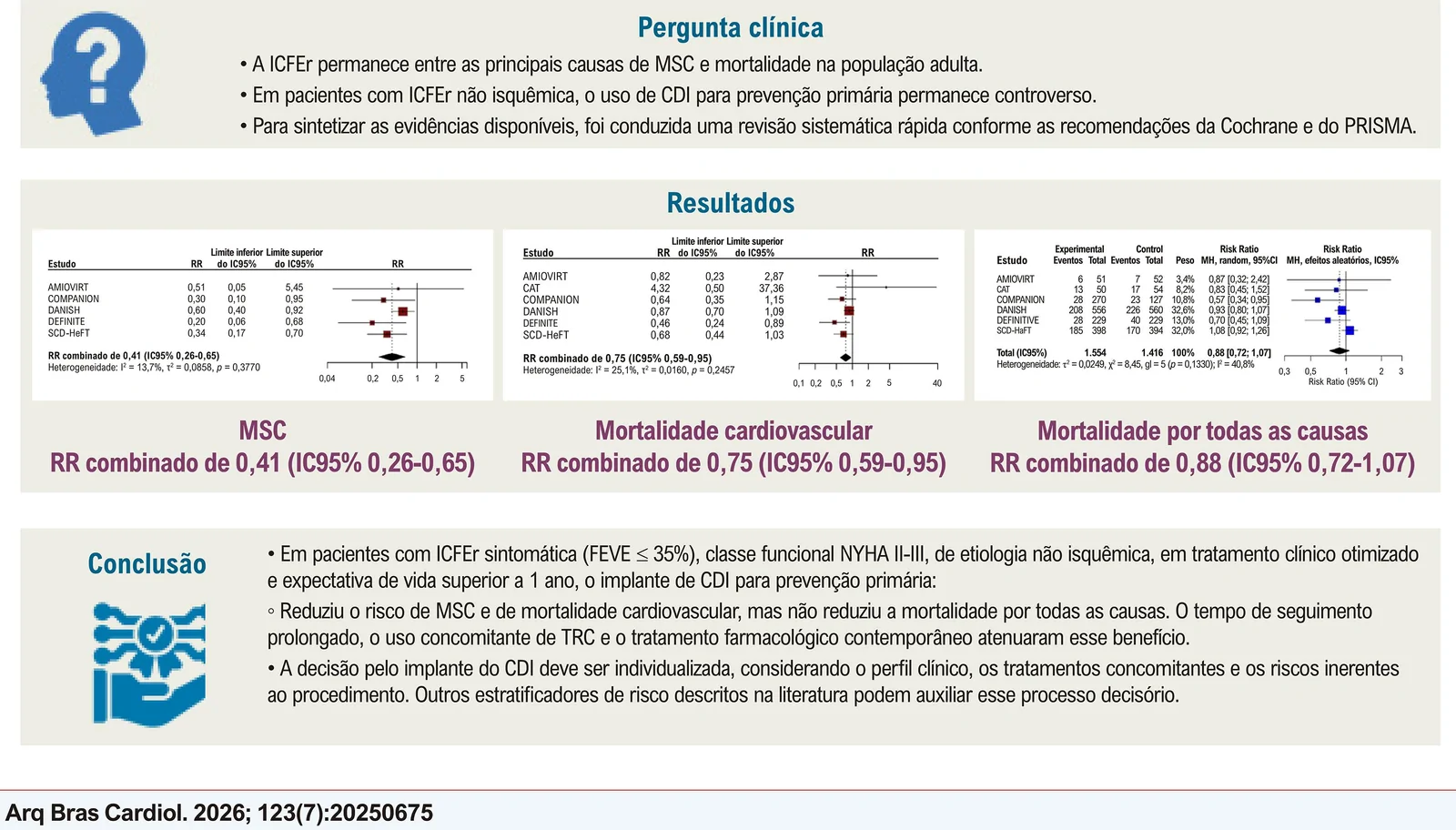
Eficácia de Cardiodesfibrilador Implantável na Prevenção Primária em Pacientes com Insuficiência Cardíaca com Fração de Ejeção Reduzida não Isquêmica CDI: cardiodesfibrilador implantável; FEVE: fração de ejeção do ventrículo esquerdo; IC95%: intervalo de confiança de 95%; ICFEr: insuficiência cardíaca com fração de ejeção reduzida; MSC: morte súbita cardíaca; NYHA: New York Heart Association; PRISMA: Preferred Reporting Items for Systematic Reviews and Meta-Analyses; RR: risco relativo; TRC: terapia de ressincronização cardíaca.

## Introdução

Avanços no tratamento da insuficiência cardíaca (IC) levaram a um declínio significativo das taxas absolutas de morte súbita cardíaca (MSC) nas últimas 4 décadas.^1^ Tanto as estratégias farmacológicas^2,3^ quanto aquelas baseadas em dispositivos^4^ demonstraram reduzir o risco de MSC e melhorar a sobrevida global. No entanto, a MSC ainda é responsável por aproximadamente 30%-40% dos óbitos observados em ensaios clínicos randomizados (ECRs) contemporâneos envolvendo pacientes com IC com fração de ejeção (FE) reduzida (ICFEr).^5-7^

O uso de cardiodesfibrilador implantável (CDI) constitui uma estratégia central para a prevenção primária e secundária da MSC, embora sua utilização em nível mundial permaneça muito inferior à recomendada pelas diretrizes clínicas.^8^ Assim, o impacto da terapia com CDI sobre a sobrevida, particularmente em pacientes com cardiomiopatia não isquêmica, passou a ser questionado após a publicação do ensaio DANISH,^9^ que demonstrou redução do risco de MSC, porém sem benefício na mortalidade por todas as causas.

Desde então, especialistas em IC têm discutido em quais cenários clínicos o implante de CDI deve ser indicado em pacientes com cardiomiopatias não isquêmicas, buscando identificar fatores adicionais para estratificação do risco de MSC. Idade avançada,^10^ presença de fibrose miocárdica^11^ e genótipos de risco^12^ têm sido apontados como características relevantes para subsidiar a decisão pelo implante do CDI, visando otimizar o equilíbrio entre benefício clínico, riscos e custos.

Com o objetivo de esclarecer o benefício do implante de CDI para a prevenção da MSC em pacientes com ICFEr não isquêmica, considerando as melhores evidências científicas disponíveis, o Departamento de Insuficiência Cardíaca da Sociedade Brasileira de Cardiologia desenvolveu a presente metanálise sobre o tema.

## Métodos

### Delineamento do estudo

Esta revisão sistemática rápida seguiu as recomendações do Cochrane Rapid Reviews Methods Group^13^ e o *checklist* Preferred Reporting Items for Systematic Reviews and Meta-Analyses.^14^ O protocolo foi registrado na plataforma PROSPERO.

### Variáveis de interesse

A questão de revisão foi estruturada segundo o modelo PICO. A população (P) de interesse compreendeu pacientes com ICFEr não isquêmica (FE do ventrículo esquerdo [FEVE] < 35%), sem histórico de arritmias ventriculares malignas, outras cardiopatias estruturais relevantes (p.ex., valvopatias) ou episódios prévios de morte súbita revertida, fibrilação ventricular ou taquicardia ventricular instável (prevenção secundária). A intervenção (I) consistiu no implante de CDI, intracavitário ou subepicárdico, comparado à ausência de implante ou ao tratamento padrão. Os desfechos (*outcomes* [O]) incluíram MSC, mortalidade cardiovascular, mortalidade por todas as causas, eventos adversos relacionados à IC e complicações associadas ao CDI, como infecção de loja do dispositivo, endocardite e necessidade de explante. Foram incluídos exclusivamente ECRs, por representarem o delineamento metodológico com maior nível de evidência para avaliação da eficácia de intervenções, minimizando vieses de seleção e fatores de confusão.

### Critérios de elegibilidade

Os critérios de inclusão foram i) ECRs envolvendo pacientes com ICFEr não isquêmica, FEVE < 35% e indicação para prevenção primária; ii) comparação direta entre CDI e tratamento padrão; e iii) relato de pelo menos um dos desfechos de interesse. Os critérios de exclusão foram i) estudos observacionais, revisões narrativas, revisões sistemáticas e editoriais; ii) estudos com populações mistas sem análise estratificada para o subgrupo de interesse; iii) estudos que avaliaram exclusivamente outros dispositivos implantáveis, como terapia de ressincronização cardíaca (TRC), marca-passos convencionais ou dispositivos de assistência ventricular; e iv) análises secundárias dos mesmos ECRs que não apresentassem novos dados.

### Busca e seleção de estudos

Inicialmente, foram realizadas buscas por revisões sistemáticas sobre o tema do presente estudo nas bases de dados MEDLINE, Embase e CENTRAL. Os detalhes da metodologia e das estratégias de busca estão descritos no material suplementar (Tabelas S1 e S2). As buscas foram realizadas em 7 de março de 2025, sem restrição de idioma ou ano de publicação.

A seleção dos estudos foi conduzida utilizando o Rayyan^15^ para remoção de duplicatas e triagem inicial. Após essa etapa, os registros remanescentes foram avaliados por um revisor, com validação por um segundo revisor. A avaliação dos textos completos e a extração dos dados seguiram o mesmo procedimento.

Foi elaborado um formulário padronizado para extração dos dados, incluindo identificação dos estudos, características dos participantes, critérios de inclusão e exclusão, descrição dos grupos de intervenção e controle, desfechos avaliados, tempo de seguimento, informações sobre financiamento e potenciais conflitos de interesse. Quando necessário, os autores dos estudos foram contatados para obtenção de dados faltantes.

### Qualidade metodológica da revisão

A identificação da revisão sistemática a ser atualizada foi realizada com base na similaridade da pergunta de revisão em relação à dúvida clínica da presente síntese de evidências e na avaliação da qualidade metodológica por meio da ferramenta AMSTAR 2.^16^

### Risco de viés e certeza da evidência dos ensaios clínicos randomizados

O risco de viés dos ECRs foi avaliado utilizando a ferramenta RoB 2, desenvolvida pela Cochrane, que examina um conjunto fixo de domínios relacionados a diferentes aspectos do desenho, da condução e do relato dos ECRs. O julgamento do risco de viés em cada domínio é gerado por algoritmo, com base nas respostas às perguntas de sinalização, podendo ser classificado como “baixo risco de viés”, “alto risco de viés” ou “algumas preocupações”.^17^

Adicionalmente, a certeza da evidência foi avaliada por meio da abordagem GRADE,^18^ que considera limitações no desenho dos estudos, risco de viés, inconsistência entre os resultados, imprecisão das estimativas, evidência indireta e possibilidade de viés de publicação. A evidência para cada desfecho foi classificada em quatro níveis: i) alta, ii) moderada, iii) baixa ou iv) muito baixa.

### Análise estatística

A síntese dos dados incluiu análise narrativa e metanálise para cada desfecho, quando havia dados disponíveis. As metanálises foram realizadas com dados dos ECRs incluídos, utilizando modelo de efeitos aleatórios no *software* RStudio, versão 2024.12.1+563. Quando necessário, dados ausentes foram imputados conforme as recomendações do Cochrane Handbook for Systematic Reviews of Interventions.^18^

Para os estudos que não apresentaram a média do tempo de seguimento, mas forneceram a mediana acompanhada do primeiro (Q1) e do terceiro (Q3) quartis, a média foi estimada pela fórmula proposta por Wan et al.^19^ Em seguida, a estimativa combinada do tempo médio de seguimento entre os estudos incluídos foi calculada por média ponderada, utilizando o tamanho amostral de cada estudo como peso, de modo a refletir adequadamente sua contribuição relativa. Nos estudos que relataram apenas a mediana, sem informações adicionais, como quartis ou valores extremos, não foi possível aplicar os métodos de estimativa previamente descritos. Nesses casos, a mediana foi considerada uma aproximação da média apenas quando o tamanho amostral era superior a 70 participantes.^19^

Na ausência do número de eventos, o número aproximado de mortes em cada grupo foi estimado com base nas taxas acumuladas de eventos apresentadas nas curvas de Kaplan-Meier.^20^ Para a análise combinada, adotou-se o risco relativo (RR) como medida de efeito. Quando o estudo reportava apenas a medida de efeito e seu intervalo de confiança, foram utilizados os valores publicados. Para combinar essas estimativas com estudos que forneciam apenas o número de eventos e o total de participantes em cada braço, os valores de RR calculados foram transformados em log(RR), com o respectivo erro padrão, para inclusão na metanálise.

Nos estudos que reportaram *hazard ratios* (HRs), essas estimativas foram incluídas nas análises combinadas como medidas relativas de efeito, analisadas em escala logarítmica natural por métodos de variância inversa. Foi adotado intervalo de confiança de 95% (IC95%).

O teste *Q* de Cochran foi utilizado para detectar heterogeneidade, considerando-se p < 0,05 como estatisticamente significativo. Complementarmente, foi calculada o I^2^ de Higgins para quantificar a proporção da variação total atribuível à heterogeneidade entre os estudos. A interpretação do I^2^ seguiu os seguintes critérios: 0%-40%, heterogeneidade possivelmente não relevante; 30%-60%, heterogeneidade possivelmente moderada; 50%-90%, heterogeneidade possivelmente substancial; e 75%-100%, heterogeneidade possivelmente considerável.^18^

O viés de publicação foi avaliado por meio de *funnel plots* e dos testes de Egger e Begg, quando o número de estudos permitiu. Também foram realizadas análises de sensibilidade para os desfechos primários, com a exclusão de estudos que apresentavam pelo menos um domínio classificado como de alto risco de viés ou com algumas preocupações.

## Resultados

### Seleção dos estudos

Na primeira etapa da busca, voltada à identificação de revisões sistemáticas, foram identificadas 88 referências nas bases de dados. Após a remoção de quatro registros duplicados, 84 referências foram submetidas à triagem por título e resumo. Destas, cinco foram selecionadas para leitura na íntegra e, após avaliação, três revisões sistemáticas foram incluídas.^21-23^ (Figura S1). A avaliação da qualidade metodológica, realizada por meio da ferramenta AMSTAR 2, demonstrou limitações significativas em duas dessas revisões,^22,23^ enquanto a terceira foi classificada como de alta qualidade metodológica^21^ (Tabela 3S).

Na busca por ECRs, foram identificados 461 registros. Após a remoção de 41 registros duplicados e a exclusão de 347 estudos com base na triagem por título e resumo, 73 referências foram selecionadas para leitura na íntegra. Adicionalmente, um registro foi identificado por busca manual. Ao final do processo de seleção, 18 publicações foram incluídas na revisão sistemática, das quais 12 correspondiam a seguimentos de seis ECRs principais. Destas, cinco publicações foram incluídas na metanálise de MSC,^24-28^ cinco na de mortalidade cardiovascular^24-26,28,29^ e seis na de mortalidade por todas as causas^24-26,28-30^ (Figura 2S). Publicações mais recentes dos estudos incluídos que avaliaram os desfechos de interesse também foram identificadas durante a busca. A relação dos estudos excluídos e suas respectivas justificativas encontra-se apresentada na Tabela 4S.

### Características dos estudos incluídos

Foram extraídos dados de seis ECRs, cujas características estão apresentadas na Tabela 1. Os estudos foram conduzidos entre 1991 e 2020 e todos apresentaram delineamento multicêntrico, envolvendo centros localizados nos Estados Unidos,^24,28-32^ Alemanha,^29^ Dinamarca,^9^ Israel,^30^ Canadá^31^e Nova Zelândia.^33^ O número total de participantes com cardiomiopatia não isquêmica incluídos foi de 3.674, com idade média variando de 52 ± 11 anos^27^ a 63 ± 12,6 anos.^32^ A FEVE média foi semelhante entre os estudos, variando de 21,4%^30^ a 25%.^9^ Dos seis ECRs incluídos, dois compararam o implante de CDI ao uso de amiodarona ou placebo,^24,32^ enquanto os demais utilizaram cuidado padrão ou placebo como grupo controle.^9,28-30,32^ O tempo de seguimento variou de 1 a 12 anos.

**Tabela 1:** – Características dos ECRs incluídos

Características	AMIOVIRT^24^	CAT^29^	COMPANION^32^	DANISH^9,26^	DEFINITE^27,30^	SCD-HeFT^4,28,31^
Delineamento do estudo	ECR, não cego	ECR	ECR, não cego	ECR, não cego, controlado e de grupos paralelos	ECR iniciado por investigadores, fundamentado em dados observacionais	ECR prospectivo de prevenção primária
Local	Estados Unidos (10 centros)	Alemanha (15 centros)	Estados Unidos (128 centros)	Dinamarca (cinco centros)	Estados Unidos e Israel (48 centros)	Canadá, Nova Zelândia e Estados Unidos (148 centros)
Período e tempo de seguimento	Período: agosto de 1996 a junho de 2001. Tempo médio de seguimento: 24,0 ± 15,6 meses.	Período: julho de 1991 a março de 1997. Tempo médio de seguimento: - Mortalidade por todas as causas (incidência em 5 anos): 66,0 ± 26,4 meses. - Mortalidade cardiovascular (incidência em 1 ano): 22,8 ± 4,3 meses.	Período: janeiro de 2000 a dezembro de 2002. Tempo mediano de seguimento: - Mortalidade por todas as causas: 17,4 meses no grupo TRC-M, 16,9 meses no grupo TRC-D e 16,0 meses no grupo controle.	Período: dezembro de 2007 a junho de 2016 (estudo principal). O estudo de extensão prolongou o seguimento até maio de 2020. Tempo mediano de seguimento: 9,5 anos (P25-P75: 7,9-10,9 anos).	Período: 2002 a 2004. Tempo médio de seguimento: 29,0 ± 14,4 meses.	Período: setembro de 1997 a julho de 2001. Tempo médio de seguimento: - Mortalidade por todas as causas: 11,0 ± 1,63 anos. - MSC e mortalidade cardiovascular: 45,5 meses.
Participantes	n = 103 (70% do sexo masculino). Idade: 59,0 ± 11,5 anos. Características clínicas: FEVE média de 23%; duração média da cardiomiopatia dilatada não isquêmica de 3,2 anos; 83% dos pacientes em classe NYHA II ou NYHA III.	n = 104 (78,9% do sexo masculino). Idade: 52,0 ± 11,0 anos. Características clínicas: FEVE média de 24%; 65,3% dos pacientes em classe NYHA II e 34,6% em classe NYHA III; 96% utilizavam IECA.	n total = 1.520 n (não isquêmicos) = 682 (51,0% do sexo masculino). Idade: 63 ± 12,6 anos. Características clínicas: FEVE mediana de 20%; 88% dos pacientes em classe NYHA III; duração mediana da IC de 3,8 anos; 41% com diabetes melito.	n = 1.116 (72,0% do sexo masculino). Idade: 63,5 ± 12,2 anos. Características clínicas: FEVE média de 25%; 53% dos pacientes em classe NYHA II, 45% em NYHA III e 1% em NYHA IV; duração média da IC de 1,5 ano; 19% com diabetes melito. Pacientes com marca-passo pré-existente ou TRC também puderam ser incluídos caso aceitassem uma possível atualização do dispositivo.	n = 458 (71,2% do sexo masculino). Idade: 58,3 ± 47,1 anos*. Características clínicas: FEVE média de 21,4%; 21,6% dos pacientes em classe funcional NYHA I, 57,4% em NYHA II e 21,0% em NYHA III; duração média da IC de 2,83 anos; 22,9% com diabetes melito.	n total = 2.521 n (não isquêmicos) = 792 (77,0% do sexo masculino). Idade: 60,0 ± 12,5 anos. Características clínicas: FEVE média de 25%; 70% dos pacientes em classe funcional NYHA II; 29% com diabetes melito, 17% com doença pulmonar, 52% com hipercolesterolemia e 56% com HAS.
Intervenção	Amiodarona, iniciada na dose de 800 mg/dia e reduzida para 300 mg/dia após 1 ano. n = 52	Implante de CDI. n = 50	(1) Terapia farmacológica otimizada + TRC-M. n = 617 n (não isquêmicos) = 285. (2) Terapia farmacológica otimizada + TRC-D. n = 595 n (não isquêmicos) = 270.	Implante de CDI associado à terapia medicamentosa otimizada. n = 556	Implante de CDI de câmara única. n = 229	(1) Amiodarona: dose de ataque de 800 mg/dia por 1 semana, seguida de 400 mg/dia por 3 semanas e posterior ajuste da dose de manutenção conforme o peso corporal. n = 419 (2) Implante de CDI de câmara única programado para modo somente choque. n = 398
Comparador	Implante de CDI. n = 51 Ambos os grupos receberam terapia medicamentosa concomitante com IECA, betabloqueadores e diuréticos poupadores de potássio.	Tratamento padrão. n = 54 Ambos os grupos receberam terapia medicamentosa concomitante com IECA e betabloqueadores.	Terapia farmacológica otimizada composta por IECA, bloqueadores dos receptores da angiotensina, betabloqueadores e espironolactona, conforme tolerado, além de diuréticos quando necessário. Digoxina e outros medicamentos para IC puderam ser utilizados a critério do investigador. n = 308 n (não isquêmicos) = 127.	Tratamento clínico usual, sem CDI, incluindo IECA ou bloqueador do receptor de angiotensina e betabloqueador. n = 560	Tratamento médico padrão, incluindo IECA, betabloqueadores e outras terapias conforme indicação clínica. n = 229	Placebo administrado com o mesmo esquema da amiodarona. n = 394 Todos os grupos receberam terapia médica convencional, incluindo betabloqueadores, IECA, antagonistas da aldosterona, ácido acetilsalicílico e estatinas, quando clinicamente indicados.
Desfechos^1^ e momento de avaliação	Desfecho primário: mortalidade por todas as causas. Desfechos secundários: MSC, morte não súbita de causa cardíaca, morte não cardíaca, síncope, sobrevida livre de arritmias, qualidade de vida e custos. Momento de avaliação: acompanhamento a cada 4 meses.	Desfecho primário: mortalidade por todas as causas. Desfechos secundários: transplante cardíaco, mortalidade de causa cardíaca (subdividida em súbita e não súbita), taquicardia ventricular sustentada e taquiarritmias ventriculares sintomáticas que exigiram tratamento antiarrítmico. Momento de avaliação: acompanhamento a cada 3 meses.	Desfecho primário: mortalidade ou hospitalização por todas as causas. Desfechos secundários: mortalidade por todas as causas, mortalidade ou hospitalização por causas cardiovasculares, mortalidade ou hospitalização por IC, eventos relacionados ao dispositivo e eventos relacionados à condição clínica do paciente. Momento de avaliação: da randomização até a ocorrência do primeiro evento.	Desfecho primário: mortalidade por todas as causas. Desfechos secundários: MSC, morte cardiovascular, parada cardíaca ressuscitada, taquicardia ventricular sustentada, alterações na qualidade de vida e infecção relacionada ao dispositivo. Momento de avaliação: aos 2 meses e, posteriormente, a cada 6 meses.	Desfecho primário: mortalidade por todas as causas. Desfecho secundário: morte súbita de origem arrítmica. Momento de avaliação: acompanhamento a cada 3 meses.	Desfecho primário: mortalidade por todas as causas. Desfechos secundários: MSC, morte arrítmica e hospitalização por IC congestiva. Momento de avaliação: acompanhamento a cada 3 meses.
Fontes de financiamento	Guidant	Guidant	Guidant	Medtronic e outros financiadores	St. Jude Medical	National Heart, Lung, and Blood Institute; National Institutes of Health; Medtronic; Wyeth-Ayerst Laboratories; Knoll Pharmaceuticals.

^1^Todos os estudos adotaram nível de significância de 5% para o DESFECHO primário. CDI: cardiodesfibrilador implantável; ECR: ensaio clínico randomizado; FEVE: fração de ejeção do ventrículo esquerdo; HAS: hipertensão arterial sistêmica; IC: insuficiência cardíaca; IECA: inibidor da enzima conversora da angiotensina; MSC: morte súbita cardíaca; NYHA: New York Heart Association; TRC: terapia de ressincronização cardíaca; TRC-D: TRC com desfibrilador; TRC-M: TRC com marca-passo.

### Morte súbita cardíaca

Foram incluídos quatro estudos que apresentaram dados brutos do número de eventos e avaliaram o efeito do CDI sobre a ocorrência de MSC em pacientes com ICFEr: AMIOVIRT, COMPANION, DANISH e DEFINITE.^24-27^ A metanálise desses estudos demonstrou que, após um seguimento médio de 5,7 anos, o CDI promoveu redução significativa do risco de MSC em comparação ao grupo controle (RR 0,44; IC95% 0,24-0,80; alta certeza da evidência; Figura S3).

O estudo SCD-HeFT não apresentou o número de eventos para os pacientes com cardiomiopatia não isquêmica em sua publicação original, mas reportou a medida de efeito correspondente em uma subanálise publicada posteriormente.^28^ A inclusão desses dados manteve a direção dos resultados e aumentou a precisão da estimativa, evidenciando redução significativa do risco de MSC (RR 0,41; IC95% 0,26-0,65; alta certeza da evidência) (Figura 1A).

**Figura 1:**
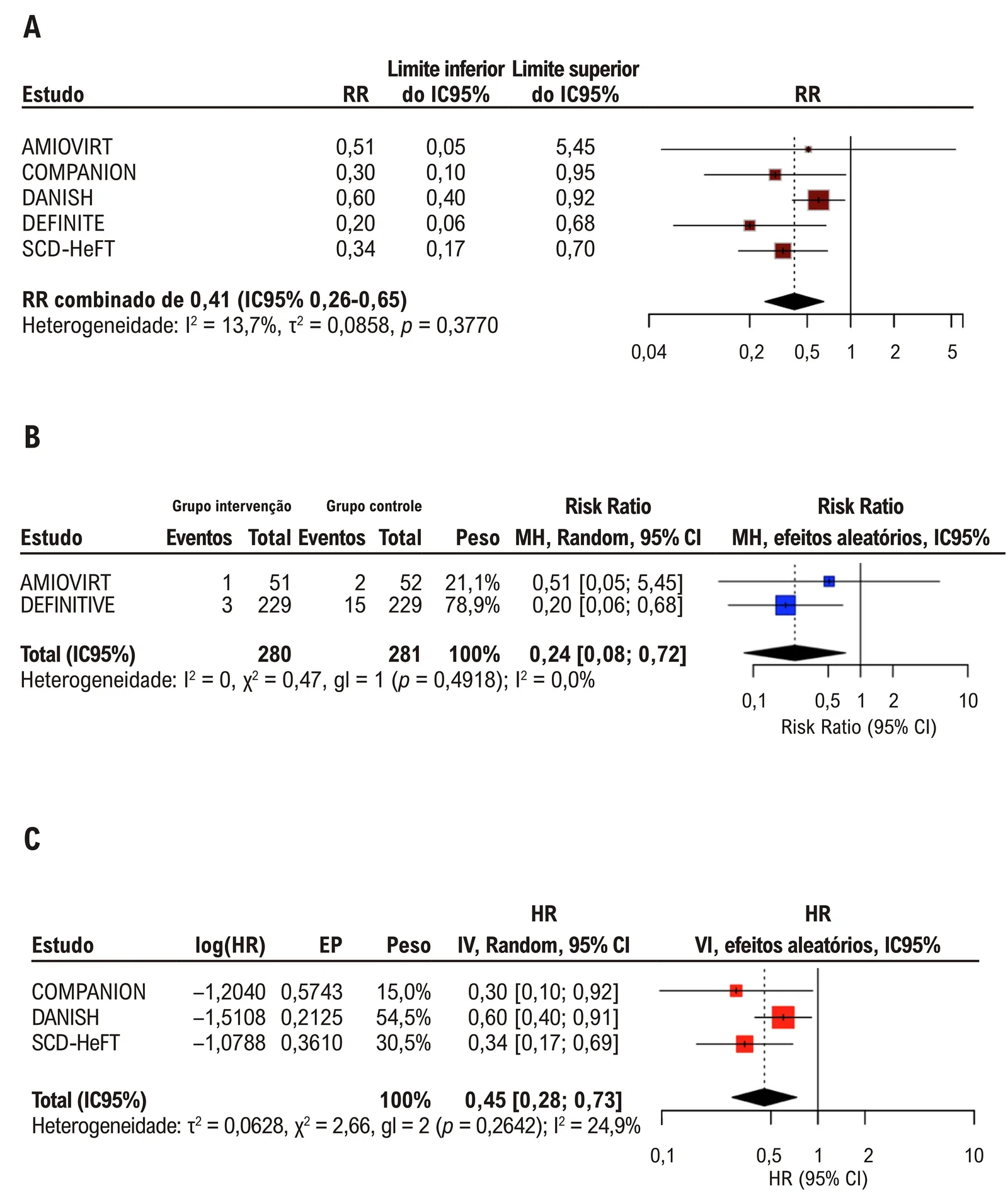
– Forest plots para o desfecho MSC. A) Análise principal, combinando os estudos por meio das estimativas de efeito disponíveis. Para os estudos AMIOVIRT e DEFINITE, o RR foi calculado a partir do número de eventos e do número total de participantes em cada grupo; para COMPANION, DANISH e SCD-HeFT, foram utilizadas as estimativas de efeito reportadas pelos autores. B) Análise de sensibilidade incluindo apenas estudos que reportaram RRs. C) Análise de sensibilidade incluindo apenas estudos que reportaram HRs. EP: erro padrão; gl: graus de liberdade; HR: hazard ratio; IC95%: intervalo de confiança de 95%; MH: método de Mantel-Haenszel; MSC: morte súbita cardíaca; RR: risco relativo; VI: variância inversa.

Foram realizadas análises de sensibilidade estratificadas de acordo com as diferentes medidas de efeito reportadas nos estudos originais (Figura 1B; Figura 1C).

A análise de subgrupo para avaliar a influência do risco de viés demonstrou redução do RR de MSC em todos os estratos analisados. Apesar das diferenças na magnitude do efeito, o benefício do CDI permaneceu consistente, independentemente da classificação do risco de viés dos estudos incluídos (Figura S4).

### Morte cardiovascular

Cinco ECRs relataram dados sobre mortalidade cardiovascular em pacientes com ICFEr não isquêmica.^24-26,28-30^ Na análise principal, o uso do CDI não foi associado à redução estatisticamente significativa do risco de morte cardiovascular (RR 0,76; IC95% 0,55-1,07), com evidência de certeza moderada devido à imprecisão.

O estudo SCD-HeFT não apresentou o número de eventos estratificados entre pacientes com etiologia isquêmica e não isquêmica em sua publicação original, porém reportou a medida de efeito correspondente em publicação subsequente.^28^ A inclusão desses dados resultou em redução estatisticamente significativa do risco de morte cardiovascular (RR 0,75; IC95% 0,59-0,95). A Figura 2 apresenta os resultados de ambas as análises.

**Figura 2:**
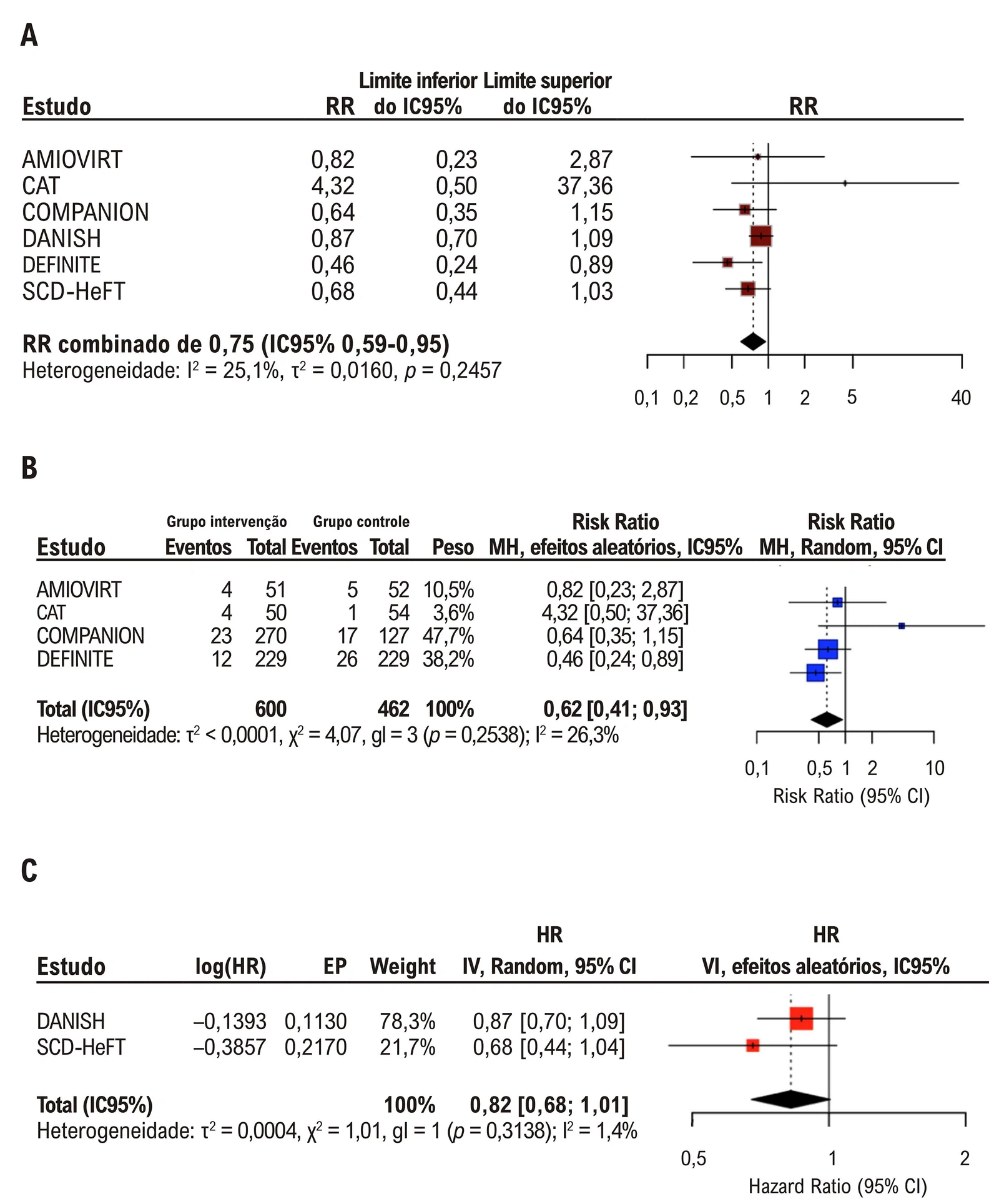
– Forest plots para o desfecho mortalidade cardiovascular. A) Análise principal, combinando os estudos por meio das estimativas de efeito disponíveis. Para os estudos AMIOVIRT, CAT, COMPANION e DEFINITE, o RR foi calculado a partir do número de eventos e do número total de participantes em cada grupo; para DANISH e SCD-HeFT, foram utilizadas as estimativas de efeito reportadas pelos autores. B) Análise de sensibilidade incluindo apenas estudos que reportaram RRs. C) Análise de sensibilidade incluindo apenas estudos que reportaram HRs. EP: erro padrão; gl: graus de liberdade; HR: hazard ratio; IC95%: intervalo de confiança de 95%; MH: método de Mantel-Haenszel; RR: risco relativo; VI: variância inversa.

Foram realizadas análises de sensibilidade estratificadas de acordo com as diferentes medidas de efeito reportadas nos estudos originais (Figura 2B; Figura 2C). Também foi conduzida análise de subgrupo para avaliar a influência do risco de viés, cujos resultados são apresentados na Figura S6.

### Mortalidade por todas as causas

Seis ECRs avaliaram o impacto do CDI sobre a mortalidade por todas as causas em pacientes com ICFEr não isquêmica: AMIOVIRT, CAT, COMPANION, DANISH, DEFINITE e SCD-HeFT.^24-26,29-31^ A análise principal não demonstrou benefício estatisticamente significativo do implante de CDI, com evidência de imprecisão nas estimativas. O risco absoluto de mortalidade foi de 341 por 1.000 pacientes no grupo controle, correspondendo a uma redução absoluta de 41 eventos por 1.000 pacientes com o uso do CDI (IC95% 24-96), com RR de 0,88 (IC95% 0,72-1,07) (Figura 3).

**Figura 3:**
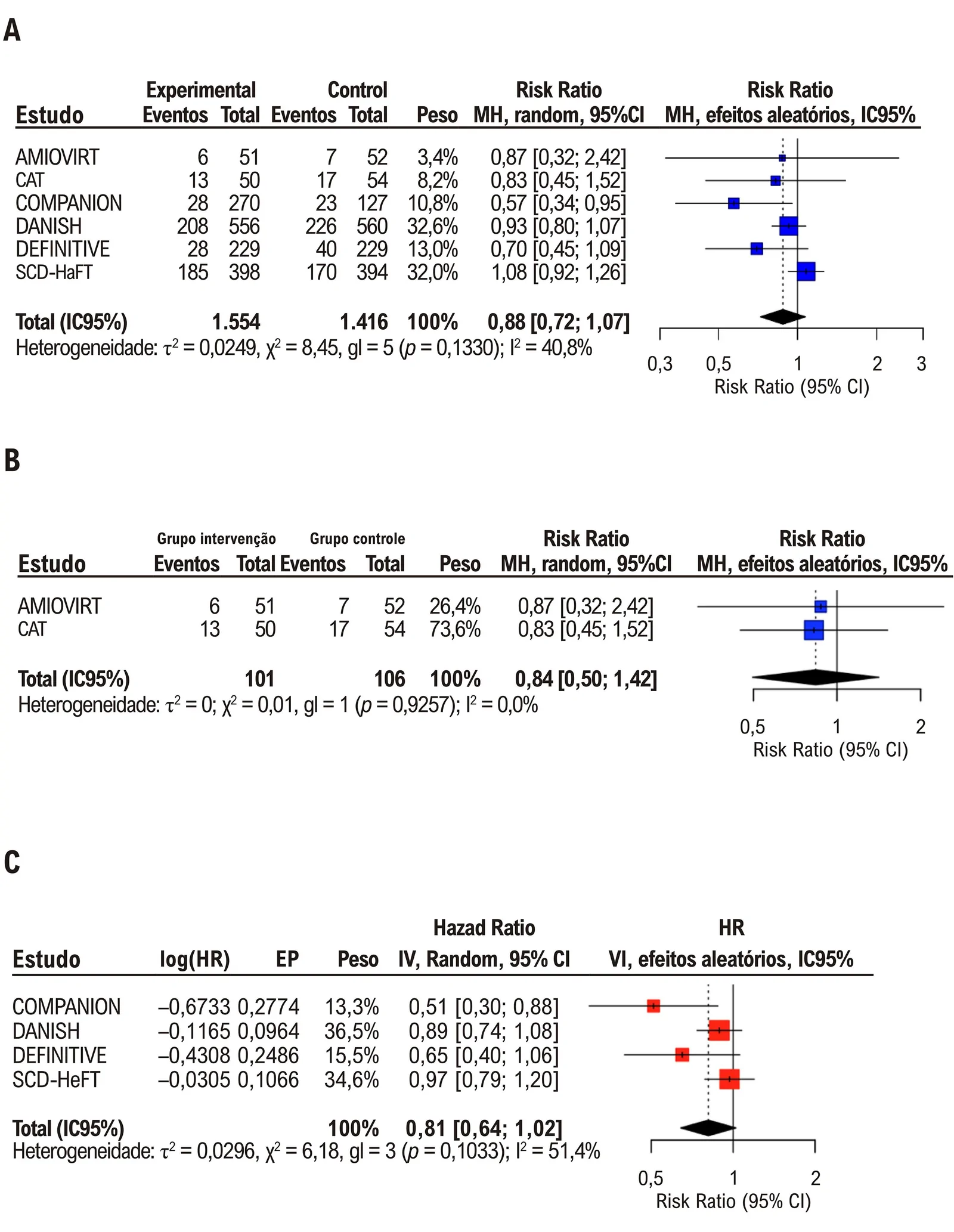
– Forest plots para o desfecho mortalidade por todas as causas. A) Análise principal, combinando os estudos por meio do número de eventos e do número total de participantes em cada grupo. B) Análise de sensibilidade incluindo apenas estudos que reportaram RRs. C) Análise de sensibilidade incluindo apenas estudos que reportaram HRs. EP: erro padrão; gl: graus de liberdade; HR: hazard ratio; IC95%: intervalo de confiança de 95%; MH: método de Mantel-Haenszel; RR: risco relativo; VI: variância inversa.

Foram realizadas análises de sensibilidade estratificadas conforme as diferentes medidas de efeito reportadas nos estudos originais (Figura 3B; Figura 3C). Em análise complementar baseada em estimativas de RR, também não foi observada diferença estatisticamente significativa (RR 0,84; IC95% 0,71-1,00; I^2^ = 19,4%; p = 0,29) (Figura S7).

Análises exploratórias foram conduzidas para melhor compreender a influência do tempo de seguimento e do uso concomitante da TRC sobre os resultados. Quando foi considerada apenas a publicação original do estudo SCD-HeFT,^33^ em substituição ao seguimento de maior duração utilizado na análise principal, o CDI foi associado à redução significativa da mortalidade por todas as causas em comparação ao grupo controle (RR 0,81; IC95% 0,69-0,95; I^2^ = 1,5%) (Figura 4). Esses achados sugerem que a inclusão da publicação de seguimento do SCD-HeFT influencia substancialmente a magnitude do efeito observado e a heterogeneidade da metanálise.

**Figura 4:**
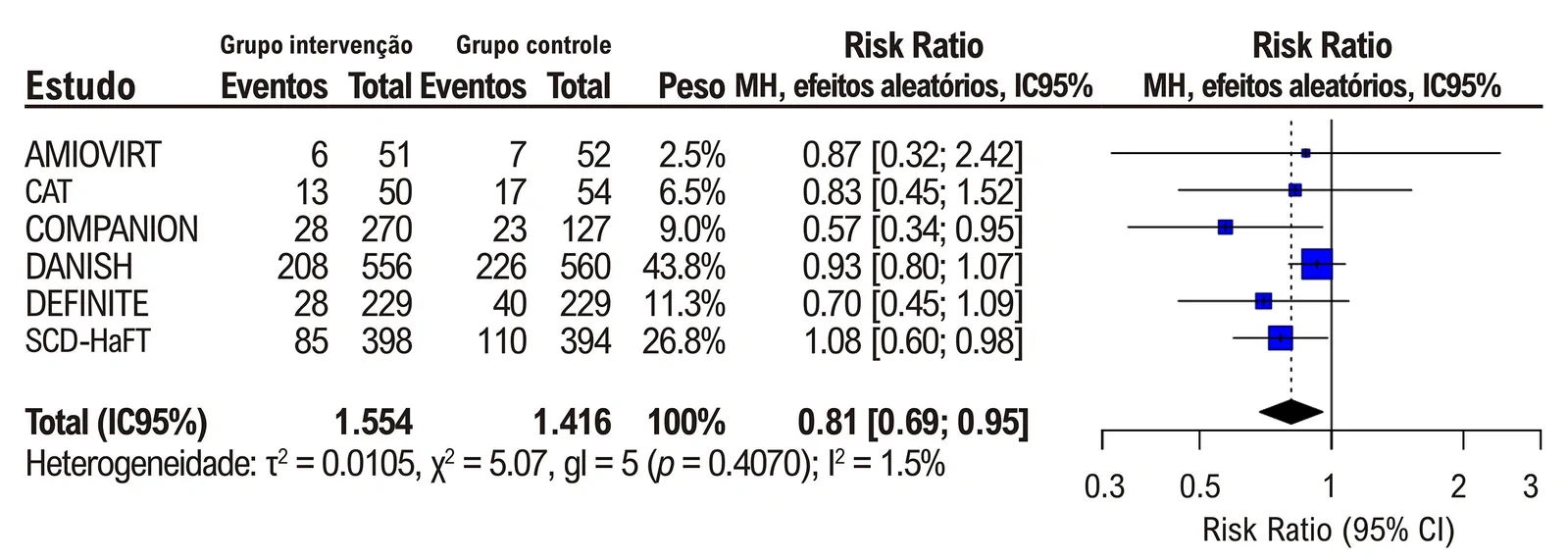
– Forest plot para o desfecho mortalidade por todas as causas utilizando os dados da publicação original do estudo SCD-HeFT. gl: graus de liberdade; HR: hazard ratio; IC95%: intervalo de confiança de 95%; MH: método de Mantel-Haenszel; RR: risco relativo.

Em análise adicional, utilizando os dados da publicação original do estudo DANISH,^9^ que permitiram incluir apenas pacientes sem TRC, e considerando simultaneamente a publicação original do SCD-HeFT,^33^ observou-se redução significativa da mortalidade por todas as causas com o uso do CDI em comparação ao grupo controle (RR 0,78; IC95% 0,66-0,91; I^2^ = 0%) (Figura 5).

**Figura 5:**
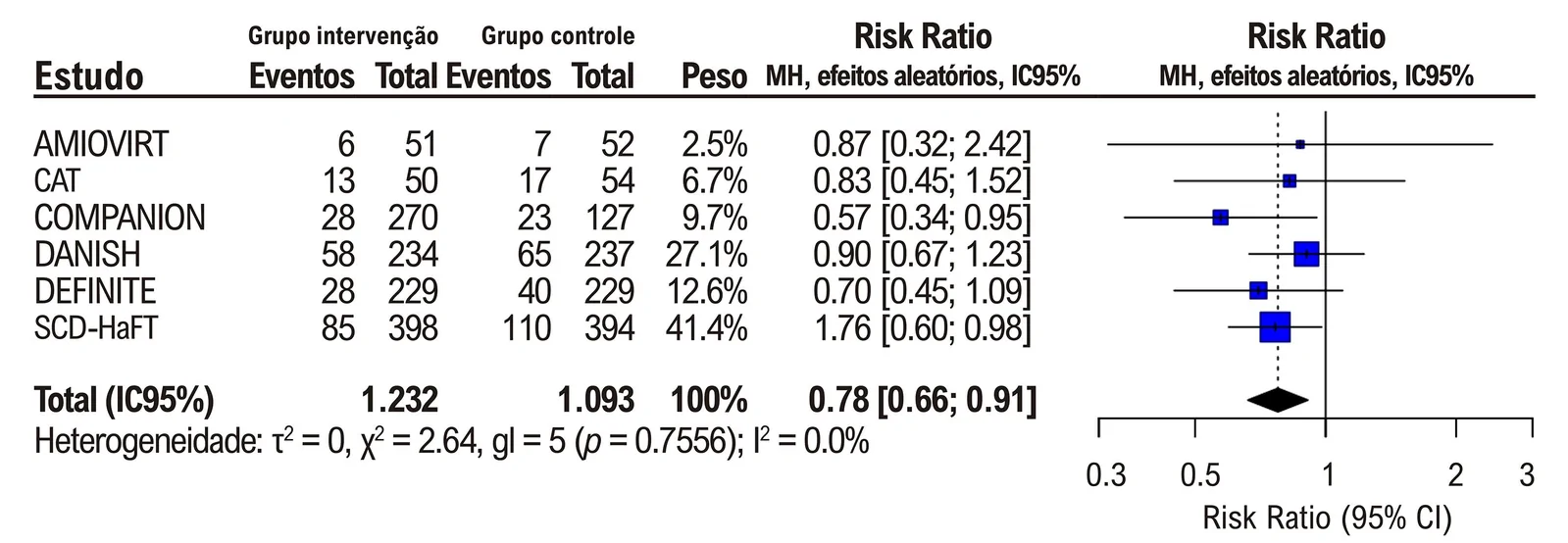
– Forest plot para o desfecho mortalidade por todas as causas utilizando os dados da publicação original dos estudos DANISH e SCD-HeFT. gl: graus de liberdade; HR: hazard ratio; IC95%: intervalo de confiança de 95%; MH: método de Mantel-Haenszel; RR: risco relativo.

Também foi realizada análise de subgrupo para avaliar a influência do risco de viés, cujos resultados são apresentados na Figura S8.

Por fim, a análise de subgrupo segundo o tempo médio de seguimento demonstrou diferença significativa nos efeitos do CDI sobre a mortalidade por todas as causas. Nos estudos com seguimento inferior a 6,8 anos,^24,25,29,30^ o CDI esteve associado à redução estatisticamente significativa do RR de mortalidade, sem evidência de heterogeneidade (RR 0,69; IC95% 0,52-0,92; I^2^ = 0%). Em contraste, não foi observada redução significativa da mortalidade por todas as causas nos estudos com seguimento superior a 6,8 anos.^26,31^ O teste de interação entre os subgrupos indicou diferença estatisticamente significativa (p = 0,02), sugerindo que a duração do seguimento pode modificar o efeito do CDI sobre esse desfecho. Além disso, complicações associadas ao uso prolongado do dispositivo e menor adesão ao acompanhamento podem contribuir para atenuar esse benefício (Figura S9).

### Complicações relacionadas ao implante e eventos relacionados à insuficiência cardíaca

Os desfechos relacionados às complicações do CDI e aos eventos relacionados à IC não foram combinados em metanálises devido à disponibilidade limitada de dados e à heterogeneidade entre os estudos. Assim, os resultados foram sintetizados de forma narrativa (Tabela 5S).

As complicações relacionadas ao implante do CDI foram relatadas em diferentes estudos, com destaque para infecção do dispositivo, choques inapropriados e complicações mecânicas. A infecção do dispositivo foi observada em dois estudos (incidência de 4,0% no estudo CAT e de 4,9% no estudo DANISH).^9,29^ No estudo DANISH, infecções graves do dispositivo, definidas como aquelas que exigiram extração do eletrodo ou representaram risco à vida, ocorreram em 2,7% dos pacientes.^9^ Entre as complicações mecânicas, foram registrados perfuração cardíaca (2% no estudo CAT)^29^ e pneumotórax (2% no estudo DANISH).^9^

As taxas de choques inapropriados foram reportadas em dois estudos, correspondendo a 5,9% no DANISH^9^ e 21,4% no DEFINITE.^30^ A incidência de deslocamento do eletrodo ou falha de detecção/isolamento foi de 14% no estudo CAT.^29^ Além disso, o estudo DEFINITE relatou complicações relacionadas ao procedimento de implante, incluindo um caso de hemotórax, um de pneumotórax e um de tamponamento cardíaco^30^ (Tabela 5S).

Os eventos relacionados à IC foram reportados em três estudos (AMIOVIRT, CAT e DEFINITE).^24,29,30^ A ocorrência de síncope variou de 3,9% a 17,0% no grupo CDI e de 0% a 14,9% no grupo controle. De modo geral, a síncope foi um evento relativamente frequente em alguns estudos, porém sem um padrão consistente de maior risco associado ao implante do CDI (Tabela 6S).

### Risco de viés e certeza da evidência

A ferramenta RoB 2 foi aplicada aos diferentes desfechos de interesse. Como todos os estudos receberam a mesma classificação de risco de viés para os desfechos avaliados, os resultados foram apresentados de forma consolidada em uma única figura (Figura S11).

A avaliação mostrou que o processo de randomização foi considerado de baixo risco em três estudos (CAT, SCD-HeFT e DANISH), enquanto apresentou algumas preocupações nos estudos AMIOVIRT, DEFINITE e COMPANION. Apesar da ausência de cegamento, não foram identificados desvios das intervenções que pudessem influenciar os desfechos, resultando em baixo risco de viés nesse domínio. Apenas o estudo COMPANION apresentou alto risco de viés devido a dados ausentes, enquanto os demais foram classificados como de baixo risco. A mensuração dos desfechos foi considerada de baixo risco em todos os estudos. Em relação ao relato seletivo dos resultados, três estudos apresentaram protocolos previamente publicados e consistentes com os resultados reportados, dois não informaram protocolo previamente registrado e um (DEFINITE) apresentou algumas preocupações quanto à seleção dos resultados apresentados.

Não foi possível realizar a avaliação formal do viés de publicação por meio dos testes de Egger e Begg, em razão do número reduzido de estudos incluídos em cada metanálise (< 10 estudos). Os *funnel plots* foram elaborados apenas para fins ilustrativos e estão apresentados na Figura S10.

A certeza da evidência foi avaliada por meio da abordagem GRADE. (Tabela 2). O desfecho de MSC e mortalidade cardiovascular foram classificados como de alta certeza da evidência. O desfecho de mortalidade por todas as causas foi classificado como de moderada certeza, indicando que o efeito verdadeiro provavelmente está próximo da estimativa obtida, embora exista a possibilidade de diferenças substanciais. A redução da certeza da evidência decorreu de limitações relacionadas à imprecisão das estimativas.

**Tabela 2:** – Avaliação da certeza da evidência para o implante de CDI na prevenção primária da mortalidade em pacientes com insuficiência cardíaca com fração de ejeção reduzida não isquêmica

Desfecho	Risco no grupo controle	Efeito absoluto com CDI (IC95%)	Efeito relativo (IC95%)	Participantes (ECRs)	Certeza da evidência (GRADE)	Interpretação
MSC Seguimento médio: 5,7 anos	—	—	RR 0,41 (0,26-0,65)	Cinco ECRs	⨁⨁⨁⨁ Alta	O CDI reduz o risco de MSC.
Mortalidade cardiovascular Seguimento médio: 5,3 anos	—	—	RR 0,75 (0,59-0,95)	Seis ECRs	⨁⨁⨁⨁ Alta	O CDI reduz a mortalidade cardiovascular.
Mortalidade por todas as causas Seguimento médio: 6,8 anos	341 por 1.000	41 eventos a menos por 1.000 (de 96 a menos a 24 a mais)	RR 0,88 (0,72-1,07)	Ao todo, 2.970 participantes (seis ECRs)	⨁⨁⨁⊝^a^ Moderada	O CDI provavelmente reduz a mortalidade por todas as causas.

*O efeito absoluto foi estimado a partir do risco observado no grupo controle e do efeito relativo da intervenção. ^a^A certeza da evidência foi reduzida em um nível devido à imprecisão, uma vez que o intervalo de confiança incluiu tanto benefício clinicamente importante quanto ausência de efeito. CDI: cardiodesfibrilador implantável; ECR: ensaio clínico randomizado; IC95%: intervalo de confiança de 95%; MSC: morte súbita cardíaca; RR: risco relativo.

## Discussão

Nesta revisão sistemática com metanálise, o implante de CDI em pacientes com ICFEr não isquêmica foi associado à redução do risco de MSC, com alta certeza da evidência, e da mortalidade cardiovascular, com moderada certeza da evidência. Em contrapartida, o benefício sobre a mortalidade por todas as causas mostrou-se menos consistente, dependendo em parte do tempo de seguimento dos pacientes. As análises também identificaram a ocorrência de complicações relacionadas ao implante, incluindo infecção do dispositivo, pneumotórax e choques inapropriados, além de eventos relacionados à IC, como síncope, com frequências variáveis entre os estudos. Esses achados reforçam que o implante do CDI não deve ser recomendado de forma indiscriminada para pacientes com ICFEr não isquêmica.

As análises de subgrupo demonstraram que o benefício do CDI sobre a mortalidade por todas as causas foi mais pronunciado em estudos com tempo de seguimento igual ou inferior a 6,8 anos, enquanto não foi observado efeito estatisticamente significativo nos estudos com acompanhamento mais prolongado. Esse ponto de corte foi definido com base no tempo médio de seguimento dos estudos incluídos nesta revisão, superior ao utilizado na revisão Cochrane (36 meses),^21^ refletindo a incorporação de publicações mais recentes. Esses achados sugerem que a duração do seguimento pode influenciar a magnitude do efeito do CDI, possivelmente em decorrência da progressão da ICFEr e do aumento da participação de causas de morte não arrítmicas e não cardiovasculares, que não são prevenidas pelo dispositivo.

Outro aspecto relevante refere-se ao uso concomitante de TRC em alguns dos estudos incluídos, especialmente DANISH^9^ e COMPANION.^32^ No estudo COMPANION, todos os pacientes do grupo de intervenção receberam TRC associada ao CDI;^32^ no estudo DANISH, aproximadamente 58% dos participantes de ambos os grupos utilizaram TRC.^9^ Considerando que a TRC reduz mortalidade e hospitalizações em pacientes com disfunção ventricular esquerda e distúrbios de condução, sua utilização pode ter atenuado o benefício incremental do CDI. No COMPANION, os efeitos observados não podem ser atribuídos exclusivamente ao CDI, dificultando a interpretação isolada de seu impacto.^32^ No estudo DANISH, por sua vez, o uso extensivo da TRC pode ter contribuído para a ausência de benefício significativo do CDI sobre a mortalidade por todas as causas, uma vez que os efeitos da TRC podem ter reduzido ou sobreposto parte do benefício esperado do desfibrilador.^9^ Além disso, o impacto do CDI sobre a mortalidade por todas as causas pode ser menos evidente na prática clínica contemporânea em razão do envelhecimento da população e da ampla incorporação de terapias farmacológicas modificadoras de prognóstico, incluindo os inibidores do receptor de angiotensina-neprilisina, os inibidores do cotransportador sódio-glicose tipo 2 e a própria TRC. Entretanto, na análise complementar realizada neste estudo, observou-se redução da mortalidade tanto entre pacientes com quanto sem TRC.

Ao comparar os achados da presente revisão com os resultados da revisão sistemática conduzida por El Moheb et al.,^21^ observa-se importante convergência quanto à eficácia do CDI. Naquela revisão, foi demonstrada redução significativa tanto da mortalidade por todas as causas (HR 0,78; IC95% 0,66-0,92) quanto da MSC (HR 0,45; IC95% 0,29-0,70). A presente revisão incorporou publicações mais recentes dos ECRs previamente analisados e realizou análises estratificadas por desfecho, risco de viés e tempo de seguimento, permitindo uma interpretação mais refinada dos resultados e a identificação de potenciais modificadores de efeito. A menor magnitude do benefício sobre a mortalidade por todas as causas observada nesta metanálise pode ser explicada, ao menos em parte, pela inclusão de estudos com seguimento mais prolongado, nos quais o aumento do risco competitivo de causas não arrítmicas tende a reduzir o impacto relativo do CDI sobre a mortalidade global. Apesar dessas diferenças, ambas as revisões sustentam o benefício do CDI na prevenção de eventos arrítmicos fatais em pacientes com ICFEr não isquêmica. Da mesma forma, ambas sugerem um possível benefício sobre a mortalidade por todas as causas, embora esse efeito permaneça sujeito a incertezas estatísticas e diferenças metodológicas entre os estudos. Permanecem, portanto, ressalvas quanto à aplicabilidade desse benefício em seguimentos prolongados, reforçando a necessidade de avaliação individualizada da relação risco-benefício para indicação do dispositivo.

Neste cenário, torna-se essencial considerar outros potenciais estratificadores de risco de MSC na prática clínica. Embora amplamente utilizada, a FE apresenta capacidade preditiva limitada quando empregada como único critério para indicação de CDI, especialmente na ICFEr não isquêmica. A presença de fibrose miocárdica, avaliada por ressonância magnética cardíaca com realce tardio, tem se mostrado um marcador robusto de risco arrítmico. Uma metanálise de 29 estudos, envolvendo aproximadamente 3.000 pacientes, demonstrou forte associação entre fibrose miocárdica e eventos arrítmicos.^33^ Além disso, coortes recentes sugerem que a presença de fibrose prediz o risco de eventos arrítmicos de forma mais acurada do que a FEVE isoladamente, e que modelos que combinam a presença de fibrose com diferentes faixas de FEVE apresentam desempenho superior ao uso exclusivo da FEVE.^11^ Outros marcadores associados ao aumento do risco incluem diâmetro diastólico final do ventrículo esquerdo superior a 73 mm, presença de ventilação periódica ao teste cardiopulmonar de exercício, taquicardia ventricular não sustentada com mais de 10 bpm ao Holter e níveis elevados de peptídeos natriuréticos.^34^ Mais recentemente, a avaliação genética também passou a desempenhar papel relevante na estratificação de risco. Variantes patogênicas de alto risco, particularmente nos genes *LMNA*, *PLN*, *FLNC*, *DSP* e *RBM20*, estão associadas a maior risco de MSC, inclusive em pacientes com FEVE > 35%. A identificação dessas variantes permite individualizar a indicação do CDI e direcionar o rastreamento familiar.^12^ Entretanto, embora sejam reconhecidos por consensos internacionais, esses marcadores ainda não foram validados como critérios de inclusão em ECRs de implante de CDI em pacientes com ICFEr.

Outro aspecto que merece consideração refere-se ao acesso desigual aos dispositivos e à sua custo-efetividade, tanto no Brasil quanto em outros países. Estudos brasileiros demonstram razões de custo-efetividade incremental elevadas para o implante de CDI, embora esses valores possam tornar-se aceitáveis quando o dispositivo é indicado após adequada estratificação de risco.^35^

### Limitações do estudo

Esta revisão sistemática apresenta algumas limitações que devem ser consideradas na interpretação dos resultados. Primeiramente, a utilização de HRs como aproximação do RRs em parte das análises, embora respaldada por recomendações metodológicas, pode introduzir imprecisão nas estimativas combinadas. Além disso, a heterogeneidade clínica entre os estudos, especialmente quanto ao tempo de seguimento, aos critérios de indicação do CDI e às características basais das populações, pode ter influenciado os resultados. A redução do benefício observada em estudos com seguimento superior a 6,8 anos pode refletir o aumento do risco competitivo de outras causas de morte, o envelhecimento das coortes e/ou a maior utilização de terapias contemporâneas modificadoras de prognóstico, devendo essa hipótese ser interpretada de forma exploratória, sem implicar relação causal.

Não foi realizada metarregressão devido ao número reduzido de estudos incluídos, condição que implicaria baixo poder estatístico e maior probabilidade de associações espúrias. Embora a heterogeneidade estatística tenha sido baixa ou moderada na maioria das análises, diferenças clínicas não mensuradas, como distintas gerações de CDI utilizadas ao longo dos estudos, podem limitar a aplicabilidade dos achados. Por fim, outra limitação relevante refere-se à escassez de dados detalhados sobre complicações relacionadas ao CDI e eventos associados à IC que, na maioria dos estudos, foram reportados de forma descritiva, o que impossibilita sua síntese quantitativa.

## Conclusão

Os achados desta revisão sistemática sustentam o benefício do CDI na prevenção primária da MSC e da mortalidade cardiovascular em pacientes com ICFEr não isquêmica. Entretanto, não foi observada redução estatisticamente significativa da mortalidade por todas as causas. O tempo de seguimento prolongado, o risco competitivo de mortes não cardiovasculares, o uso concomitante de TRC e a evolução do tratamento farmacológico contemporâneo parecem atenuar esse benefício. Dessa forma, os resultados não sustentam a indicação rotineira de CDI para todos os pacientes sintomáticos com ICFEr não isquêmica em tratamento clínico otimizado (Figura Central). A decisão pelo implante do CDI deve ser individualizada, considerando o perfil clínico do paciente, os tratamentos concomitantes e os riscos inerentes ao procedimento. Além disso, outros estratificadores de risco, já descritos na literatura científica, podem contribuir para uma seleção mais precisa dos pacientes com maior probabilidade de benefício.

## Material suplementar

Material suplementar Para informação adicional, por favor, clique aqui.

## References

[1] 1. Shen L, Jhund PS, Petrie MC, Claggett BL, Barlera S, Cleland JGF, et al. Declining Risk of Sudden Death in Heart Failure. N Engl J Med. 2017;377(1):41-51. doi: 10.1056/NEJMoa1609758. 10.1056/NEJMoa1609758 28679089

[2] 2. Al-Gobari M, El Khatib C, Pillon F, Gueyffier F. ß-Blockers for the Prevention of Sudden Cardiac Death in Heart Failure Patients: A Meta-Analysis of Randomized Controlled Trials. BMC Cardiovasc Disord. 2013;13:52. doi: 10.1186/1471-2261-13-52. 10.1186/1471-2261-13-52 PMC3716800 23848972

[3] 3. Rossello X, Ariti C, Pocock SJ, Ferreira JP, Girerd N, McMurray JJV, et al. Impact of Mineralocorticoid Receptor Antagonists on the Risk of Sudden Cardiac Death in Patients with Heart Failure and Left-Ventricular Systolic Dysfunction: An Individual Patient-Level Meta-Analysis of Three Randomized-Controlled Trials. Clin Res Cardiol. 2019;108(5):477-86. doi: 10.1007/s00392-018-1378-0. 10.1007/s00392-018-1378-0 30264282

[4] 4. Bardy GH, Lee KL, Mark DB, Poole JE, Packer DL, Boineau R, et al. Amiodarone or an Implantable Cardioverter-Defibrillator for Congestive Heart Failure. N Engl J Med. 2005;352(3):225-37. doi: 10.1056/NEJMoa043399. 10.1056/NEJMoa043399 15659722

[5] 5. Zannad F, McMurray JJ, Krum H, van Veldhuisen DJ, Swedberg K, Shi H, et al. Eplerenone in Patients with Systolic Heart Failure and Mild Symptoms. N Engl J Med. 2011;364(1):11-21. doi: 10.1056/NEJMoa1009492. 10.1056/NEJMoa1009492 21073363

[6] 6. McMurray JJ, Packer M, Desai AS, Gong J, Lefkowitz MP, Rizkala AR, et al. Angiotensin-Neprilysin Inhibition versus Enalapril in Heart Failure. N Engl J Med. 2014;371(11):993-1004. doi: 10.1056/NEJMoa1409077. 10.1056/NEJMoa1409077 25176015

[7] 7. Curtain JP, Docherty KF, Jhund PS, Petrie MC, Inzucchi SE, Køber L, et al. Effect of Dapagliflozin on Ventricular Arrhythmias, Resuscitated Cardiac Arrest, or Sudden Death in DAPA-HF. Eur Heart J. 2021;42(36):3727-38. doi: 10.1093/eurheartj/ehab560. 10.1093/eurheartj/ehab560 PMC8455345 34448003

[8] 8. Shah B, Hernandez AF, Liang L, Al-Khatib SM, Yancy CW, Fonarow GC, et al. Hospital Variation and Characteristics of Implantable Cardioverter-Defibrillator Use in Patients with Heart Failure: Data from the GWTG-HF (Get with the Guidelines-Heart Failure) Registry. J Am Coll Cardiol. 2009;53(5):416-22. doi: 10.1016/j.jacc.2008.09.045. 10.1016/j.jacc.2008.09.045 19179199

[9] 9. Køber L, Thune JJ, Nielsen JC, Haarbo J, Videbæk L, Korup E, et al. Defibrillator Implantation in Patients with Nonischemic Systolic Heart Failure. N Engl J Med. 2016;375(13):1221-30. doi: 10.1056/NEJMoa1608029. 10.1056/NEJMoa1608029 27571011

[10] 10. Elming MB, Nielsen JC, Haarbo J, Videbæk L, Korup E, Signorovitch J, et al. Age and Outcomes of Primary Prevention Implantable Cardioverter-Defibrillators in Patients with Nonischemic Systolic Heart Failure. Circulation. 2017;136(19):1772-80. doi: 10.1161/CIRCULATIONAHA.117.028829. 10.1161/CIRCULATIONAHA.117.028829 28877914

[11] 11. Di Marco A, Brown PF, Bradley J, Nucifora G, Claver E, Frutos F, et al. Improved Risk Stratification for Ventricular Arrhythmias and Sudden Death in Patients with Nonischemic Dilated Cardiomyopathy. J Am Coll Cardiol. 2021;77(23):2890-905. doi: 10.1016/j.jacc.2021.04.030. 10.1016/j.jacc.2021.04.030 34112317

[12] 12. Wilde AAM, Semsarian C, Márquez MF, Shamloo AS, Ackerman MJ, Ashley EA, et al. European Heart Rhythm Association (EHRA)/Heart Rhythm Society (HRS)/Asia Pacific Heart Rhythm Society (APHRS)/Latin American Heart Rhythm Society (LAHRS) Expert Consensus Statement on the State of Genetic Testing for Cardiac Diseases. Europace. 2022;24(8):1307-67. doi: 10.1093/europace/euac030. 10.1093/europace/euac030 PMC9435643 35373836

[13] 13. Garritty C, Gartlehner G, Nussbaumer-Streit B, King VJ, Hamel C, Kamel C, et al. Cochrane Rapid Reviews Methods Group Offers Evidence-Informed Guidance to Conduct Rapid Reviews. J Clin Epidemiol. 2021;130:13-22. doi: 10.1016/j.jclinepi.2020.10.007. 10.1016/j.jclinepi.2020.10.007 PMC7557165 33068715

[14] 14. Page MJ, McKenzie JE, Bossuyt PM, Boutron I, Hoffmann TC, Mulrow CD, et al. The PRISMA 2020 Statement: An Updated Guideline for Reporting Systematic Reviews. BMJ. 2021;372:n71. doi: 10.1136/bmj.n71. 10.1136/bmj.n71 PMC8005924 33782057

[15] 15. Ouzzani M, Hammady H, Fedorowicz Z, Elmagarmid A. Rayyan-a Web and Mobile App for Systematic Reviews. Syst Rev. 2016;5(1):210. doi: 10.1186/s13643-016-0384-4. 10.1186/s13643-016-0384-4 PMC5139140 27919275

[16] 16. Shea BJ, Reeves BC, Wells G, Thuku M, Hamel C, Moran J, et al. AMSTAR 2: A Critical Appraisal Tool for Systematic Reviews that Include Randomised or Non-Randomised Studies of Healthcare Interventions, or Both. BMJ. 2017;358:j4008. doi: 10.1136/bmj.j4008. 10.1136/bmj.j4008 PMC5833365 28935701

[17] 17. Higgins JPT, Thomas J, Chandler J, Cumpston M, Li T, Page MJ, et al, editor(s). Cochrane Handbook for Systematic Reviews of Interventions version 6.5 (Updated August 2024) [Internet]. London: Cochrane; 2024 [cited 2026 Jul 9]. Available from: www.cochrane.org/handbook.

[18] 18. Guyatt GH, Oxman AD, Vist GE, Kunz R, Falck-Ytter Y, Alonso-Coello P, et al. GRADE: An Emerging Consensus on Rating Quality of Evidence and Strength of Recommendations. BMJ. 2008;336(7650):924-6. doi: 10.1136/bmj.39489.470347.AD. 10.1136/bmj.39489.470347.AD PMC2335261 18436948

[19] 19. Wan X, Wang W, Liu J, Tong T. Estimating the Sample Mean and Standard Deviation from the Sample Size, Median, Range and/or Interquartile Range. BMC Med Res Methodol. 2014;14:135. doi: 10.1186/1471-2288-14-135. 10.1186/1471-2288-14-135 PMC4383202 25524443

[20] 20. Parmar MK, Torri V, Stewart L. Extracting Summary Statistics to Perform Meta-Analyses of the Published Literature for Survival Endpoints. Stat Med. 1998;17(24):2815-34. doi: 10.1002/(sici)1097-0258(19981230)17:24<2815::aid-sim110>3.0.co;2-8. 10.1002/(sici)1097-0258(19981230)17:24<2815::aid-sim110>3.0.co;2-8 9921604

[21] 21. El Moheb M, Nicolas J, Khamis AM, Iskandarani G, Akl EA, Refaat M. Implantable Cardiac Defibrillators for People with Non-Ischaemic Cardiomyopathy. Cochrane Database Syst Rev. 2018;12(12):CD012738. doi: 10.1002/14651858.CD012738.pub2. 10.1002/14651858.CD012738.pub2 PMC6517305 30537022

[22] 22. Narayanan MA, Vakil K, Reddy YN, Baskaran J, Deshmukh A, Benditt DG, et al. Efficacy of Implantable Cardioverter-Defibrillator Therapy in Patients with Nonischemic Cardiomyopathy: A Systematic Review and Meta-Analysis of Randomized Controlled Trials. JACC Clin Electrophysiol. 2017;3(9):962-70. doi: 10.1016/j.jacep.2017.02.006. 10.1016/j.jacep.2017.02.006 29759721

[23] 23. Cavalcanti R, Aboul-Hosn N, Morales G, Abdel-Latif A. Implantable Cardioverter Defibrillator for the Primary Prevention of Sudden Cardiac Death in Patients with Nonischemic Cardiomyopathy. Angiology. 2018;69(4):297-302. doi: 10.1177/0003319717710851. 10.1177/0003319717710851 28554215

[24] 24. Strickberger SA, Hummel JD, Bartlett TG, Frumin HI, Schuger CD, Beau SL, et al. Amiodarone versus Implantable Cardioverter-Defibrillator: Randomized Trial in Patients with Nonischemic Dilated Cardiomyopathy and Asymptomatic Nonsustained Ventricular Tachycardia--AMIOVIRT. J Am Coll Cardiol. 2003;41(10):1707-12. doi: 10.1016/s0735-1097(03)00297-3. 10.1016/s0735-1097(03)00297-3 12767651

[25] 25. Doran B, Mei C, Varosy PD, Kao DP, Saxon LA, Feldman AM, et al. The Addition of a Defibrillator to Resynchronization Therapy Decreases Mortality in Patients with Nonischemic Cardiomyopathy. JACC Heart Fail. 2021;9(6):439-49. doi: 10.1016/j.jchf.2021.02.013. 10.1016/j.jchf.2021.02.013 33992570

[26] 26. Yafasova A, Doi SN, Thune JJ, Nielsen JC, Haarbo J, Bruun NE, et al. Effect of Implantable Cardioverter-Defibrillators in Nonischemic Heart Failure According to Background Medical Therapy: Extended Follow-up of the DANISH Trial. J Card Fail. 2024;30(11):1411-20. doi: 10.1016/j.cardfail.2024.04.017. 10.1016/j.cardfail.2024.04.017 38750689

[27] 27. Ellenbogen KA, Levine JH, Berger RD, Daubert JP, Winters SL, Greenstein E, et al. Are Implantable Cardioverter Defibrillator Shocks a Surrogate for Sudden Cardiac Death in Patients with Nonischemic Cardiomyopathy? Circulation. 2006;113(6):776-82. doi: 10.1161/CIRCULATIONAHA.105.561571. 10.1161/CIRCULATIONAHA.105.561571 16461817

[28] 28. Packer DL, Prutkin JM, Hellkamp AS, Mitchell LB, Bernstein RC, Wood F, et al. Impact of Implantable Cardioverter-Defibrillator, Amiodarone, and Placebo on the Mode of Death in Stable Patients with Heart Failure: Analysis from the Sudden Cardiac Death in Heart Failure trial. Circulation. 2009;120(22):2170-6. doi: 10.1161/CIRCULATIONAHA.109.853689. 10.1161/CIRCULATIONAHA.109.853689 PMC2922511 19917887

[29] 29. Bänsch D, Antz M, Boczor S, Volkmer M, Tebbenjohanns J, Seidl K, et al. Primary Prevention of Sudden Cardiac Death in Idiopathic Dilated Cardiomyopathy: The Cardiomyopathy Trial (CAT). Circulation. 2002;105(12):1453-8. doi: 10.1161/01.cir.0000012350.99718.ad. 10.1161/01.cir.0000012350.99718.ad 11914254

[30] 30. Kadish A, Dyer A, Daubert JP, Quigg R, Estes NA, Anderson KP, et al. Prophylactic Defibrillator Implantation in Patients with Nonischemic Dilated Cardiomyopathy. N Engl J Med. 2004;350(21):2151-8. doi: 10.1056/NEJMoa033088. 10.1056/NEJMoa033088 15152060

[31] 31. Poole JE, Olshansky B, Mark DB, Anderson J, Johnson G, Hellkamp AS, et al. Long-Term Outcomes of Implantable Cardioverter-Defibrillator Therapy in the SCD-HeFT. J Am Coll Cardiol. 2020;76(4):405-15. doi: 10.1016/j.jacc.2020.05.061. 10.1016/j.jacc.2020.05.061 32703511

[32] 32. Bristow MR, Saxon LA, Boehmer J, Krueger S, Kass DA, De Marco T, et al. Cardiac-Resynchronization Therapy with or without an Implantable Defibrillator in Advanced Chronic Heart Failure. N Engl J Med. 2004;350(21):2140-50. doi: 10.1056/NEJMoa032423. 10.1056/NEJMoa032423 15152059

[33] 33. Di Marco A, Anguera I, Schmitt M, Klem I, Neilan TG, White JA, et al. Late Gadolinium Enhancement and the Risk for Ventricular Arrhythmias or Sudden Death in Dilated Cardiomyopathy: Systematic Review and Meta-Analysis. JACC Heart Fail. 201;5(1):28-38. doi: 10.1016/j.jchf.2016.09.017. 10.1016/j.jchf.2016.09.017 28017348

[34] 34. Pimentel M, Zimerman A, Chemello D, Giaretta V, Andrades M, Silvello D, et al. Predictors of Serious Arrhythmic Events in Patients with Nonischemic Heart Failure. J Interv Card Electrophysiol. 2017;48(2):131-9. doi: 10.1007/s10840-016-0213-7. 10.1007/s10840-016-0213-7 27943114

[35] 35. Ribeiro RA, Stella SF, Zimerman LI, Pimentel M, Rohde LE, Polanczyk CA. Cost-Effectiveness of Implantable Cardioverter Defibrillators in Brazil in the Public and Private Sectors. Arq Bras Cardiol. 2010;95(5):577-86. doi: 10.1590/s0066-782x2010005000134. 10.1590/s0066-782x2010005000134 20922266

