# Supplementary material for: Eficácia de Cardiodesfibrilador Implantável na Prevenção Primária em Pacientes com Insuficiência Cardíaca com Fração de Ejeção Reduzida não Isquêmica
Source: Arq Bras Cardiol. 2026 Oct 1;123(7):e20250675. [Article in Portuguese] doi: 10.36660/abc.20250675 (PMC13668461; doi:10.36660/abc.20250675)
Supplement: Supplementary file 1 [file 0066-782x-abc-123-7-20250675-suppl.pdf]

MATERIAL SUPLEMENTAR

Tabela 1S. Estratégia de busca bibliográfica para revisões sistemáticas.

Base de dados/ Data da busca	Estratégia de busca	Número de registros
Pubmed 07/03/2025	#1 "Defibrillators, Implantable"[Mesh] OR (Defibrillator, Implantable) OR (Implantable Defibrillator) OR (Implantable Cardioverter Defibrillator) OR (Cardioverter Defibrillator, Implantable) OR (Cardioverter Defibrillators, Implantable) OR (Defibrillator, Implantable Cardioverter) OR (Defibrillators, Implantable Cardioverter) OR (Implantable Cardioverter Defibrillators) OR (Cardioverter-Defibrillators, Implantable) OR (Cardioverter-Defibrillator, Implantable) OR (Implantable Cardioverter-Defibrillator) OR (Implantable Cardioverter-Defibrillators) OR (Implantable Defibrillators) OR (implantable cardioverterdefibrillator)<br><br>#2 "Heart Failure, Systolic"[Mesh] OR (Heart Failures, Systolic) OR (Systolic Heart Failures) OR (Heart Failure, Reduced Ejection Fraction) OR (Systolic Heart Failure) OR (reduced ejection fraction) OR (Heart Failure, Systolic)<br><br>#3 (((systematic review[ti] OR systematic literature review[ti] OR systematic scoping review[ti] OR systematic narrative review[ti] OR systematic qualitative review[ti] OR systematic evidence review[ti] OR systematic quantitative review[ti] OR systematic meta-review[ti] OR systematic critical review[ti] OR systematic mixed studies review[ti] OR systematic mapping review[ti] OR systematic cochrane review[ti] OR systematic search and review[ti] OR systematic integrative review[ti]) NOT comment[pt] NOT (protocol[ti] OR protocols[ti])) NOT MEDLINE [subset]) OR (Cochrane Database Syst Rev[ta] AND review[pt]) OR systematic review[pt]<br><br>#1 AND #2 AND #3	47
Embase 07/03/2025	#1 'implantable cardioverter defibrillator'/exp OR ‘Defibrillators, Implantable’ OR ‘Defibrillator, Implantable’ OR ‘Implantable Defibrillator’ OR ‘Implantable Cardioverter Defibrillator’ OR ‘Cardioverter Defibrillator, Implantable’ OR ‘Cardioverter Defibrillators, Implantable’ OR ‘Defibrillator, Implantable Cardioverter’ OR ‘Defibrillators, Implantable Cardioverter’ OR ‘Implantable Cardioverter Defibrillators’ OR ‘Cardioverter-Defibrillators, Implantable’ OR ‘Cardioverter-Defibrillator, Implantable’ OR ‘Implantable Cardioverter-Defibrillator’ OR ‘Implantable Cardioverter-Defibrillators’ OR ‘Implantable Defibrillators’ OR ‘implantable cardioverterdefibrillator’<br><br>#2 'systolic heart failure'/exp OR 'heart failure with reduced ejection fraction'/exp OR ‘Heart Failure, Systolic’ OR ‘Heart Failures, Systolic’ OR ‘Systolic Heart Failures’ OR ‘Heart Failure, Reduced Ejection Fraction’ OR ‘Systolic Heart Failure’ OR ‘reduced ejection fraction’ OR ‘Heart Failure, Systolic’<br><br>#3 ‘systematic review’/exp OR ‘scoping review’/exp OR ‘meta analysis’/exp OR ‘systematic review’ OR ‘systematic literature review’ OR ‘systematic scoping review’ OR ‘systematic narrative review’ OR ‘systematic qualitative review’ OR ‘systematic evidence review’ OR ‘systematic quantitative review’ OR ‘systematic meta-review’ OR ‘systematic critical review’ OR ‘systematic mixed studies review’ OR ‘systematic	40

	mapping review' OR 'systematic cochrane review' OR 'systematic search and review' OR 'systematic integrative review'<br><br>#4 [embase]/lim NOT ([embase]/lim AND [medline]/lim)<br><br>#1 AND #2 AND #3 AND #4	
Cochrane<br>07/03/2025	#1 MeSH descriptor: [Defibrillators, Implantable] explode all trees<br><br>#2 MeSH descriptor: [Heart Failure, Systolic] explode all trees<br><br>#3 MeSH descriptor: [Cardiomyopathies] explode all trees<br><br>#1 AND (#2 OR #3)<br><br>Cochrane reviews	1
**TOTAL**		**88**

Tabela 2S. Estratégia de busca bibliográfica para ensaios clínicos randomizados.

Base de dados/ Data da busca	Estratégia de busca	Número de estudos
Pubmed 07/03/2025	#1 "Defibrillators, Implantable"[Mesh] OR (Defibrillator, Implantable) OR (Implantable Defibrillator) OR (Implantable Cardioverter Defibrillator) OR (Cardioverter Defibrillator, Implantable) OR (Cardioverter Defibrillators, Implantable) OR (Defibrillator, Implantable Cardioverter) OR (Defibrillators, Implantable Cardioverter) OR (Implantable Cardioverter Defibrillators) OR (Cardioverter-Defibrillators, Implantable) OR (Cardioverter-Defibrillator, Implantable) OR (Implantable Cardioverter-Defibrillator) OR (Implantable Cardioverter-Defibrillators) OR (Implantable Defibrillators) OR (implantable cardioverterdefibrillator)<br><br>#2 "Heart Failure, Systolic"[Mesh] OR (Heart Failures, Systolic) OR (Systolic Heart Failures) OR (Heart Failure, Reduced Ejection Fraction) OR (Systolic Heart Failure) OR (reduced ejection fraction) OR (Heart Failure, Systolic)<br><br>#3 (randomized controlled trial[Publication Type] OR (randomized[Title/Abstract] AND controlled[Title/Abstract] AND trial[Title/Abstract]))<br><br>#1 AND #2 AND #3	256
Embase 07/03/2025	#1 'implantable cardioverter defibrillator'/exp OR 'Defibrillators, Implantable' OR 'Defibrillator, Implantable' OR 'Implantable Defibrillator' OR 'Implantable Cardioverter Defibrillator' OR 'Cardioverter Defibrillator, Implantable' OR 'Cardioverter Defibrillators, Implantable' OR 'Defibrillator, Implantable Cardioverter' OR 'Defibrillators, Implantable Cardioverter' OR 'Implantable Cardioverter Defibrillators' OR 'Cardioverter-Defibrillators, Implantable' OR 'Cardioverter-Defibrillator, Implantable' OR 'Implantable Cardioverter-Defibrillator' OR 'Implantable Cardioverter-Defibrillators' OR 'Implantable Defibrillators' OR 'implantable cardioverterdefibrillator'<br><br>#2 'systolic heart failure'/exp OR 'heart failure with reduced ejection fraction'/exp OR 'Heart Failure, Systolic' OR 'Heart Failures, Systolic' OR 'Systolic Heart Failures' OR 'Heart Failure, Reduced Ejection Fraction' OR 'Systolic Heart Failure' OR 'reduced ejection fraction' OR 'Heart Failure, Systolic'<br><br>#3 'randomized controlled trial'/exp OR 'randomized controlled trial'<br><br>#4 [embase]/lim NOT ([embase]/lim AND [medline]/lim)<br><br>#1 AND #2 AND #3 AND #4	78
Cochrane 07/03/2025	#1 MeSH descriptor: [Defibrillators, Implantable] explode all trees<br><br>#2 MeSH descriptor: [Heart Failure, Systolic] explode all trees<br><br>#3 MeSH descriptor: [Cardiomyopathies] explode all trees<br><br>#1 AND (#2 OR #3)<br><br>Trials	127
**TOTAL**		**461**

Tabela 3S. Características das revisões sistemáticas selecionadas.

Autor/ Ano	Tipo de estudos	Intervenção	População	Estudos primários	Motivo para não considerar atualização da revisão
**Narayanan, M. 2017**	ECR	CDI	Pacientes com insuficiência cardíaca não isquêmica com fração de ejeção <35%	AMIOVIRT, 2003<br>CAT, 2002<br>COMPANION, 2004<br>DANISH, 2016<br>DEFINITE Trial, 2004<br>SCD-HeFT, 2005	Foram encontradas limitações metodológicas significativas avaliadas pela ferramenta AMSTAR 2. Não há menção sobre a existência de um protocolo prévio. A busca foi limitada, sem justificativas, para data e idioma Inglês, e não foi apresentada a lista com os estudos excluídos da revisão. Os autores não justificam a inclusão de ECR, assim como não reportam o financiamento dos estudos incluídos. Além disso, o risco de viés dos estudos incluídos não foi avaliado, tampouco sua influência nos resultados encontrados.
**Cavalcanti, R. 2018**	ECR	CDI	Pacientes com insuficiência cardíaca não isquêmica com fração de ejeção <35%	AMIOVIRT, 2003<br>CAT, 2002<br>DANISH, 2016<br>DEFINITE Trial, 2004<br>SCD-HeFT, 2005	Foram encontradas limitações metodológicas significativas avaliadas pela ferramenta AMSTAR 2. Os componentes da pergunta PICO não foram claramente descritos e não há menção sobre a existência ou ausência de um protocolo prévio. Também não há justificativa para a inclusão de ECR, nem análise de financiamento dos estudos incluídos. A busca foi limitada, e os autores não apresentaram a lista de estudos excluídos. Além disso, os autores conduziram a metanálise sem considerar o impacto do risco de viés dos estudos individuais nos resultados obtidos, assim como não discutiram o impacto deste viés na interpretação dos resultados.
**El Moheb, M. 2018**	ECR	CDI em adição à terapia medicamentosa otimizada	Pacientes com insuficiência cardíaca não isquêmica com fração de ejeção <35%	AMIOVIRT, 2003<br>CAT, 2002<br>COMPANION, 2004<br>DANISH, 2016<br>DEFINITE Trial, 2004<br>SCD-HeFT, 2005	Não foram encontradas limitações metodológicas significativas, no entanto, novos dados foram publicados após as análises deste estudo terem sido feitas. Portanto, uma atualização desta revisão foi realizada.

AMIOVIRT; Amiodarone versus Implantable Defibrillator. AMSTAR; A Measurement Tool to Assess Systematic Reviews. CAT; Cardiomyopathy Trial. CDI; cardiodesfibrilador implantável. COMPANION; Comparison of Medical Therapy, Pacing, and Defibrillation in Heart Failure. DANISH; The Danish Study to Assess the Efficacy of ICDs in Patients With Nonischemic Systolic Heart Failure on Mortality. DEFINETE; DEFibrillators In Non-Ischemic Cardiomyopathy Treatment Evaluation. ECR; Ensaio Clínico Randomizado. PICO; Paciente, Intervenção, Comparação e Outcome (desfecho). SCD-HeFT; Sudden Cardiac Death in Heart Failure Trial

Tabela 4S. Estudos excluídos e motivos das exclusões.

Autor / Ano	Título	Motivos para a exclusão
Namerow, P.B., 1999	Quality-of-life six months after CABG surgery in patients randomized to ICD versus no ICD therapy: findings from the CABG Patch Trial.	Ausência dos desfechos avaliados
Kadish, A., 2000	Defibrillators in nonischemic cardiomyopathy treatment evaluation	Ausência dos desfechos avaliados (protocolo do estudo)
Sheldon, R., 2000	Identification of patients most likely to benefit from implantable cardioverter-defibrillator therapy: the Canadian Implantable Defibrillator Study.	População não elegível
O'Brien, B.J., 2001	Cost-effectiveness of the implantable cardioverter-defibrillator: results from the Canadian Implantable Defibrillator Study (CIDS).	População não elegível
Steinberg, J.S., 2001	Antiarrhythmic drug use in the implantable defibrillator arm of the Antiarrhythmics Versus Implantable Defibrillators (AVID) Study.	População não elegível
Schaechter, A., 2003	DEFibrillators In Non-Ischemic Cardiomyopathy Treatment Evaluation (DEFINITE)	Ausência dos desfechos avaliados (dados da linha de base)
Cawley, P.J., 2004	Amiodarone versus implantable cardioverter defibrillator for asymptomatic nonsustained ventricular tachycardia in nonischemic dilated cardiomyopathy	Comentário
Goldberger, J.J., 2006	Effects of Statin Therapy on Arrhythmic Events and Survival in Patients With Nonischemic Dilated Cardiomyopathy	Ausência dos desfechos avaliados
Kadish, A., 2006	Patients with recently diagnosed nonischemic cardiomyopathy benefit from implantable cardioverter-defibrillators	Ausência dos desfechos avaliados
Rashba, E.J., 2006	Preserved heart rate variability identifies low-risk patients with nonischemic dilated cardiomyopathy: results from the DEFINITE trial	Ausência dos desfechos avaliados
Dickinson, M.G., 2007	Statin use was associated with reduced mortality in both ischemic and nonischemic cardiomyopathy and in patients with implantable defibrillators: mortality data and mechanistic insights from the Sudden Cardiac Death in Heart Failure Trial (SCD-HeFT).	Ausência dos desfechos avaliados

Freudenberger, R.S., 2007	Risk of thromboembolism in heart failure: an analysis from the Sudden Cardiac Death in Heart Failure Trial (SCD-HeFT).	Ausência de dados da população de interesse (não-isquêmicos)
Passman, R., 2007	Implantable cardioverter defibrillators and quality of life: results from the defibrillators in nonischemic cardiomyopathy treatment evaluation study.	Ausência dos desfechos avaliados
De Marco, T., 2008	Impact of cardiac resynchronization therapy on exercise performance, functional capacity, and quality of life in systolic heart failure with QRS prolongation: COMPANION trial sub-study.	Ausência dos desfechos avaliados
Gold, M.R., 2008	Role of microvolt T-wave alternans in assessment of arrhythmia vulnerability among patients with heart failure and systolic dysfunction: primary results from the T-wave alternans sudden cardiac death in heart failure trial substudy.	Ausência dos desfechos avaliados
Mitchell, J.E., 2008	Outcome in African Americans and other minorities in the Sudden Cardiac Death in Heart Failure Trial (SCD-HeFT).	Ausência de dados da população de interesse (não-isquêmicos)
Olshansky, B., 2008	Syncope predicts the outcome of cardiomyopathy patients: analysis of the SCD-HeFT study	Ausência dos desfechos avaliados
Russo, A.M., 2008	Primary prevention with defibrillator therapy in women: results from the Sudden Cardiac Death in Heart Failure Trial.	Ausência dos desfechos avaliados
Daubert, J.P., 2009	Ventricular arrhythmia inducibility predicts subsequent ICD activation in nonischemic cardiomyopathy patients: a DEFINITE substudy	Ausência dos desfechos avaliados
Olshansky, B., 2012	Does cardiac resynchronization therapy provide unrecognized benefit in patients with prolonged PR intervals? The impact of restoring atrioventricular synchrony: an analysis from the COMPANION Trial.	Ausência dos desfechos avaliados
Zecchin, M., 2012	How can optimization of medical treatment avoid unnecessary implantable cardioverter-defibrillator implantations in patients with idiopathic dilated cardiomyopathy presenting with "SCD-HeFT criteria?"	Ausência dos desfechos avaliados
Martinelli, M., 2013	CHronic use of Amiodarone aGAinSt Implantable cardioverter-defibrillator therapy for primary prevention of death in patients with Chagas cardiomyopathy Study: rationale and design of a randomized clinical trial	População não elegível
Mitchell, J.E., 2013	Thyroid function in heart failure and impact on mortality.	Ausência dos desfechos avaliados

Schliamser, J.E., 2013	Significance of follow-up left ventricular ejection fraction measurements in the Defibrillators in Non-Ischemic Cardiomyopathy Treatment Evaluation trial (DEFINITE).	Ausência dos desfechos avaliados
Bristow, M.R., 2016	Lessons Learned and Insights Gained in the Design, Analysis, and Outcomes of the COMPANION Trial.	Ausência dos desfechos avaliados
Elming, M.B., 2017	Age and effect of implantable cardioverter-defibrillator (ICD) in patients with non-ischemic systolic heart failure	Resumo de congresso
Elming, M.B., 2017	Myocardial fibrosis and the effect of defibrillator implantation in patients with non-ischemic systolic heart failure - DANISH-MRI	Resumo de congresso
Lin, J., 2017	Effect of PR Interval on Outcomes Following Cardiac Resynchronization Therapy: A Secondary Analysis of the COMPANION Trial.	Ausência dos desfechos avaliados
Bundgaard, J., 2018	The impact of ICD implantation on health-related quality of life in the DANISHtrial	Resumo de congresso
Elming, M.B., 2018	Right ventricular dysfunction and the effect of defibrillator implantation in patients with nonischemic systolic heart failure	Resumo de congresso
Levy, W.C., 2018	Improving the Use of Primary Prevention Implantable Cardioverter-Defibrillators Therapy With Validated Patient-Centric Risk Estimates.	Ausência dos desfechos avaliados
Bundgaard, J.S., 2019	The impact of implantable cardioverter-defibrillator implantation on health-related quality of life in the DANISH trial.	Ausência dos desfechos avaliados
Elming, M.B., 2019	Duration of Heart Failure and Effect of Defibrillator Implantation in Patients With Nonischemic Systolic Heart Failure.	Ausência dos desfechos avaliados
Elming, M.B., 2019	Right Ventricular Dysfunction and the Effect of Defibrillator Implantation in Patients With Nonischemic Systolic Heart Failure.	Ausência dos desfechos avaliados
Levy, W.C., 2019	Maximizing survival benefit with primary prevention implantable cardioverter-defibrillator therapy in a heart failure population.	Ausência dos desfechos avaliados
Lund-Andersen, C., 2019	Association between QRS shortening and mortality after implantation of cardiac resynchronization therapy: Results from the DANISH study	Resumo de congresso

Jukema, J.W., 2019	Prophylactic Use of Implantable Cardioverter-Defibrillators in the Prevention of Sudden Cardiac Death in Dialysis Patients.	População não elegível
Rørth, R., 2019	Efficacy of an implantable cardioverter-defibrillator in patients with diabetes and heart failure and reduced ejection fraction.	Ausência dos desfechos avaliados
Rørth, R., 2019	The effect of implantable cardioverter-defibrillator in patients with diabetes and non-ischaemic systolic heart failure.	Ausência dos desfechos avaliados
Shamoun, F., 2019	Impact of Degree of Left Ventricular Remodeling on Clinical Outcomes From Cardiac Resynchronization Therapy.	Ausência dos desfechos avaliados
Bundgaard, J.S., 2020	Quality of life and the associated risk of all-cause mortality in nonischemic heart failure.	Ausência dos desfechos avaliados
Elming, M.B., 2020	Myocardial fibrosis and the effect of primary prophylactic defibrillator implantation in patients with non-ischemic systolic heart failure-DANISH-MRI	Ausência dos desfechos avaliados
Jensen, D.H., 2020	Right ventricular free wall strain and effect of implantable defibrillator in non-ischemic heart failure	Resumo de congresso
Jilek, C., 2020	Cardioverter-defibrillator does not improve short-term survival among patients with non-ischemic cardiomyopathy and reduced left ventricular ejection fraction	Ausência dos desfechos avaliados
Carlson, S., 2021	Trajectory of left ventricular ejection fraction among individuals eligible for implantable cardioverter-defibrillator	Ausência dos desfechos avaliados
Boas, R., 2021	Prevalence and prognostic association of ventricular arrhythmia in non-ischaemic heart failure patients: results from the DANISH trial.	Ausência dos desfechos avaliados
Boas, R., 2022	Periodic Repolarization Dynamics Identifies ICD Responders in Nonischemic Cardiomyopathy: a DANISH Substudy	Ausência dos desfechos avaliados
Butt, J.H., 2022	NT-proBNP and ICD in Nonischemic Systolic Heart Failure: Extended Follow-Up of the DANISH Trial.	Ausência dos desfechos avaliados
Byrne, C., 2022	Effect of implantable cardioverter-defibrillators in patients with non-ischaemic systolic heart failure and concurrent coronary atherosclerosis.	Ausência dos desfechos avaliados
Doi, S.N., 2022	Diabetes may modify the efficacy of primary prevention implantable cardioverter-defibrillators in non-	Resumo de congresso

	ischemic systolic heart failure: An extended follow-up of the DANISH trial	
Eiger, D.S., 2022	Factors and outcomes associated with improved left ventricular systolic function in patients with cardiomyopathy.	Ausência dos desfechos avaliados
Yafasova, A., 2022	Cardiac resynchronisation therapy and implantable cardioverter-defibrillator in non-ischaemic systolic heart failure: Extended follow-up of the DANISH trial	Resumo de congresso
Doi, S.N., 2023	Efficacy of implantable cardioverter-defibrillator in non-ischemic systolic heart failure in patients with and without chronic kidney disease: an extended follow-up analysis of the DANISH trial	Resumo de congresso
Yafasova, A., 2023	Effect of implantable cardioverter-defibrillator in non-ischaemic systolic heart failure according to the Heart Failure Collaboratory Medical Therapy Score: extended follow-up of the DANISH trial	Resumo de congresso
Doi, S.N., 2024	Estimated Glomerular Filtration Rate and Implantable Cardioverter-Defibrillator in Nonischemic Systolic Heart Failure: Extended Follow-Up of DANISH.	Ausência dos desfechos avaliados
Segar, M.W., 2024	Frailty Status Modifies the Efficacy of ICD Therapy for Primary Prevention Among Patients With HF.	Registro não obtido

Tabela 5S. Complicações relacionadas ao implante do dispositivo.

Complicação	Eventos grupo CDI (n/total)	Eventos grupo controle (n/total)
**Infecção do dispositivo**		
DANISH Trial	27/556 (4,9%)	20/560 (3,6%)[1]
CAT	2/50 (4%)	-
**Infecção grave do dispositivo[2]**		
DANISH Trial	15/556 (2,7%)	13/560 (2,3%)[1]
**Perfuração**		
CAT	1/50 (2%)	-
**Pneumothorax**		
DANISH Trial	11/556 (2%)	6/560 (1,1%)
**Sangramento que requer intervenção**		
DANISH Trial	1/556 (2%)	0/556 (0%)
**Choques inapropriados**		
DANISH Trial	33/556 (5,9%)	-
DEFINITE	49/229 (21,4%)	-
**Deslocamento do eletrodo e defeito de detecção/isolamento**		
CAT	7/50 (14%)	-
**Complicações durante o implante do CDI**		
DEFINITE Trial		
Hemotórax	1/458 (0,2%)	-
Pneumotórax	1/458 (0,2%)	-
Tamponamento	1/458 (0,2%)	-

[1]Pacientes que já possuíam marcapasso ou que receberam um novo dispositivo após a randomização.
[2]Infecção que exige extração do eletrodo, tratamento antibiótico por toda a vida ou que cause óbito.

Tabela 6S. Eventos relacionados a insuficiência cardíaca.

Evento	Eventos grupo CDI (n/total)	Eventos grupo controle (n/total)
**Síncope**		
AMIOVIRT	2/51 (3,9%)	3/52 (5,9%)
DEFINITE Trial	39/229 (17%)	34/229 (14,9%)
CAT	6/50 (12%)	0/54 (0%)

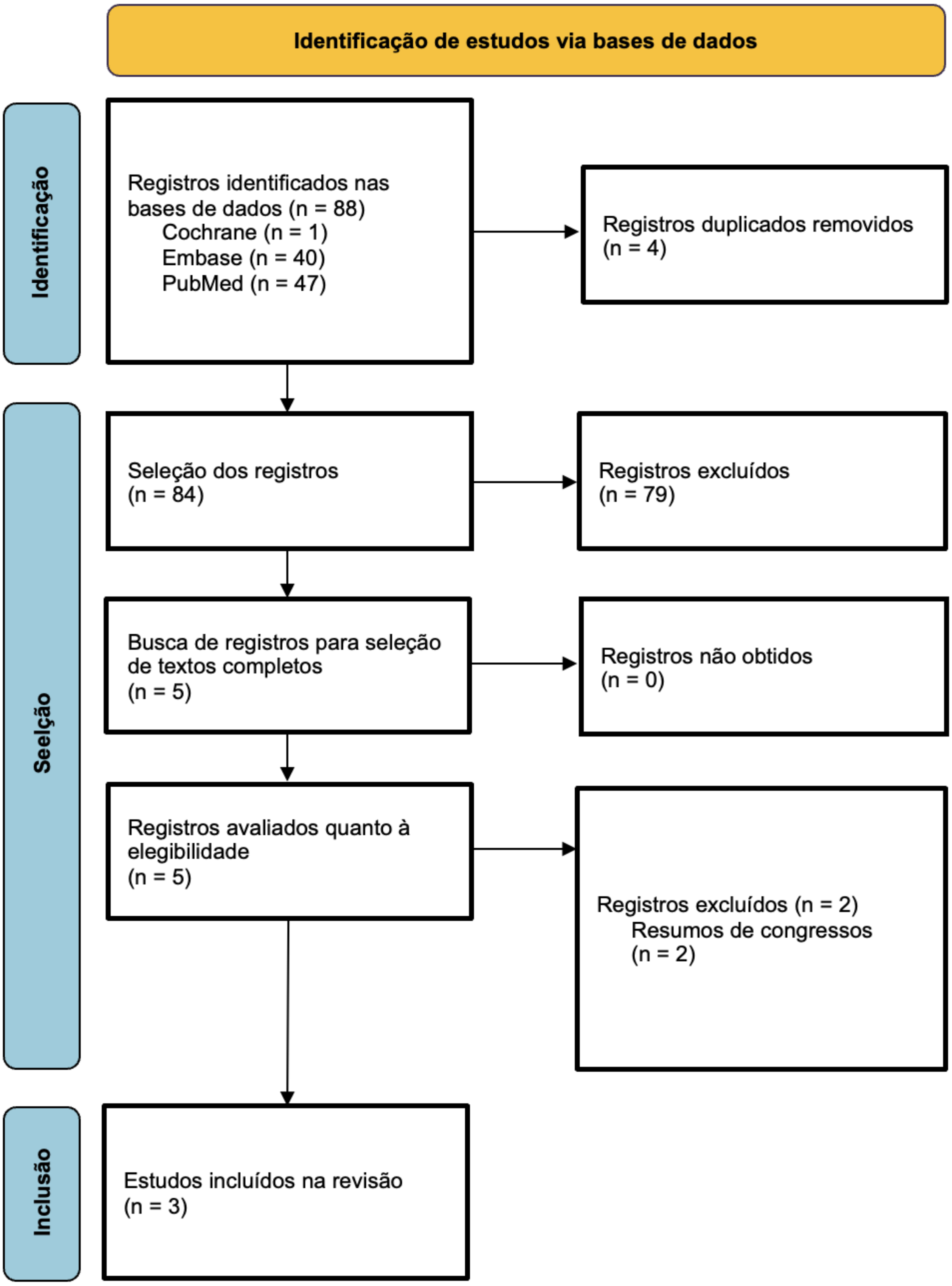

Figura 1S. Fluxograma PRISMA da busca por revisões sistemáticas.

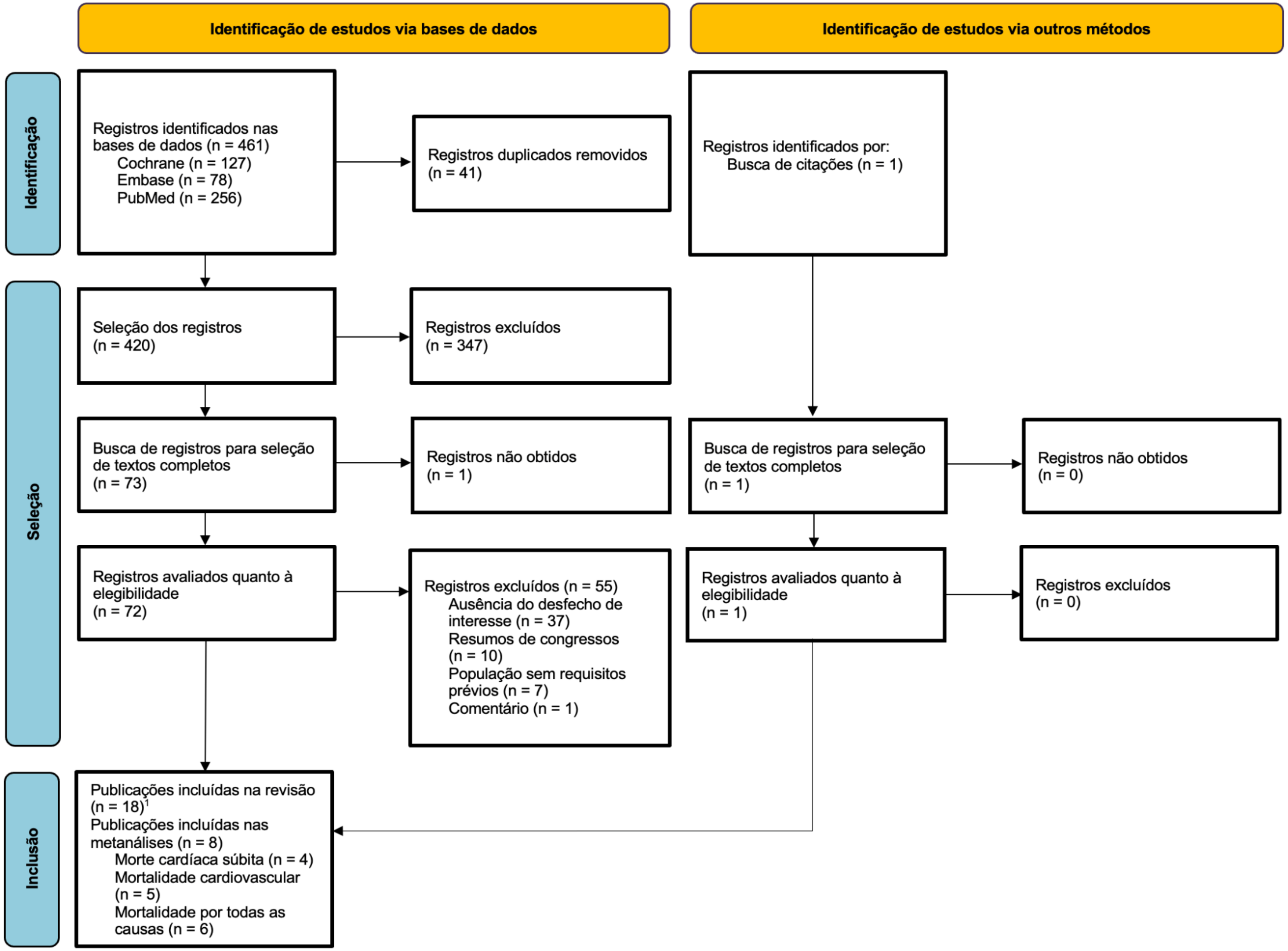

Figura 2S. Fluxograma PRISMA da busca por ensaios clínicos randomizados. [1]18 publicações oriundas de 6 estudos principais.

Study	Experimental Events	Experimental Total	Control Events	Control Total	Weight	Risk Ratio MH, Random, 95% CI
AMIOVIRT	1	51	2	52	6.1%	0.51 [0.05; 5.45]
COMPANION	5	270	7	127	20.8%	0.34 [0.11; 1.04]
DANISH	35	556	57	560	54.8%	0.62 [0.41; 0.93]
DEFINITE Trial	3	229	15	229	18.4%	0.20 [0.06; 0.68]
**Total (95% CI)**		**1106**		**968**	**100.0%**	**0.44 [0.24; 0.80]**

Heterogeneity: $Tau^2 = 0.1336$; $Chi^2 = 3.61$, df = 3 (P = 0.3063); $I^2 = 17.0\%$

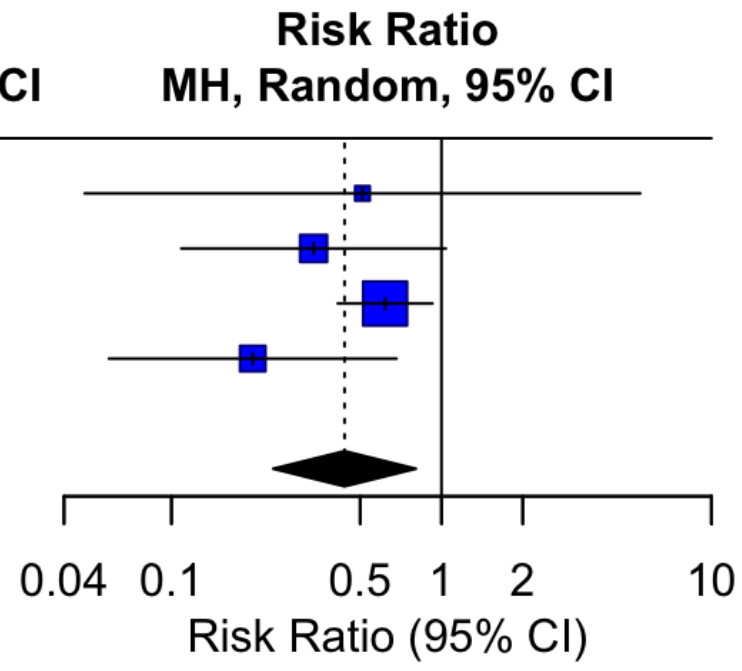

Figura 3S. Gráfico de floresta sobre morte súbita cardíaca.
Desfecho avaliado através da análise combinada do número de eventos/n total de cada estudo.

Study or Subgroup	Experimental Events	Experimental Total	Control Events	Control Total	Weight	Risk Ratio MH, Random, 95% CI
**Viés = Baixo**						
DANISH	35	556	57	560	54.8%	0.62 [0.41; 0.93]
**Viés = Algumas considerações**						
AMIOVIRT	1	51	2	52	6.1%	0.51 [0.05; 5.45]
DEFINITE Trial	3	229	15	229	18.4%	0.20 [0.06; 0.68]
**Total (95% CI)**		**280**		**281**	**24.4%**	**0.24 [0.08; 0.72]**
Heterogeneity: $Tau^2 = 0$; $Chi^2 = 0.47$, df = 1 (P = 0.4918); $I^2 = 0\%$						
**Viés = Alto**						
COMPANION	5	270	7	127	20.8%	0.34 [0.11; 1.04]
**Total (95% CI)**		**1106**		**968**	**100.0%**	**0.44 [0.24; 0.80]**

Heterogeneity: $Tau^2 = 0.1336$; $Chi^2 = 3.61$, df = 3 (P = 0.3063); $I^2 = 17.0\%$
Test for subgroup differences: $Chi^2 = 3.14$, df = 2 (P = 0.2080)

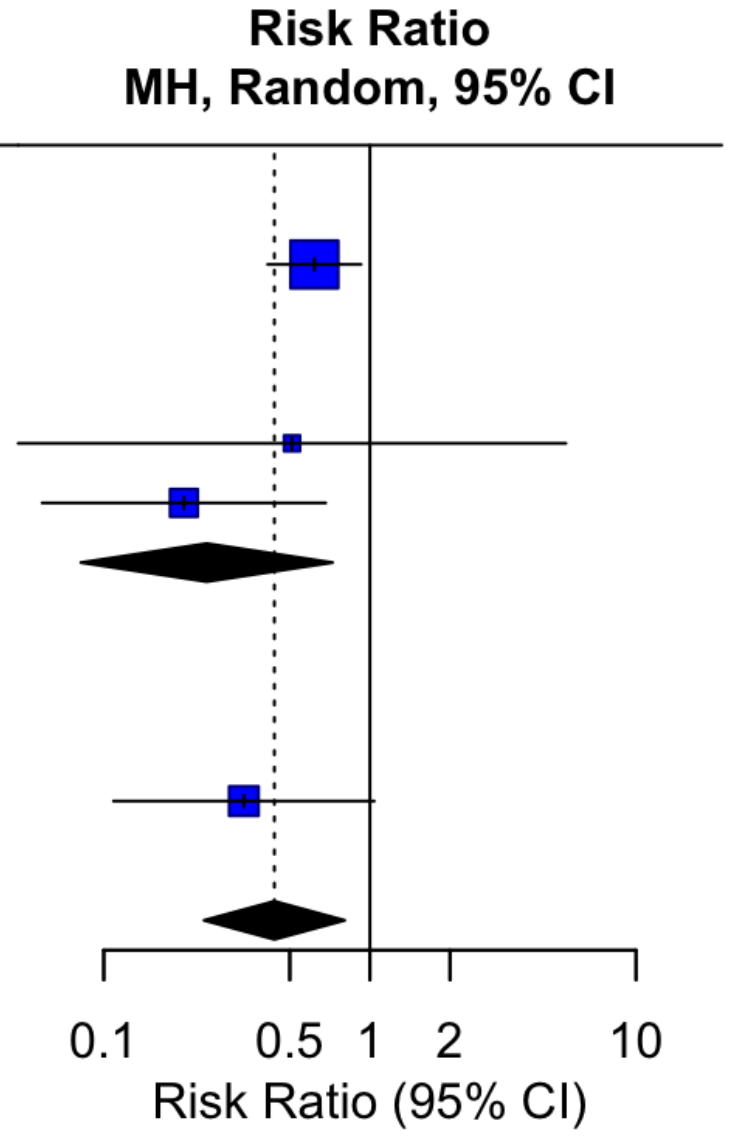

Figura 4S. Análise estratificada por avaliação do risco de viés para o desfecho morte cardíaca súbita.

Study	Experimental Events	Experimental Total	Control Events	Control Total	Weight	Risk Ratio MH, Random, 95% CI
AMIOVIRT	4	51	5	52	6.4%	0.82 [0.23; 2.87]
CAT	4	50	1	54	2.3%	4.32 [0.50; 37.36]
COMPANION	23	270	17	127	21.3%	0.64 [0.35; 1.15]
DANISH	147	556	164	560	51.6%	0.90 [0.75; 1.09]
DEFINITE Trial	12	229	26	229	18.3%	0.46 [0.24; 0.89]
**Total (95% CI)**		**1156**		**1022**	**100.0%**	**0.76 [0.55; 1.07]**

Heterogeneity: $Tau^2 = 0.0477$; $Chi^2 = 6.79$, df = 4 (P = 0.1473); $I^2 = 41.1\%$

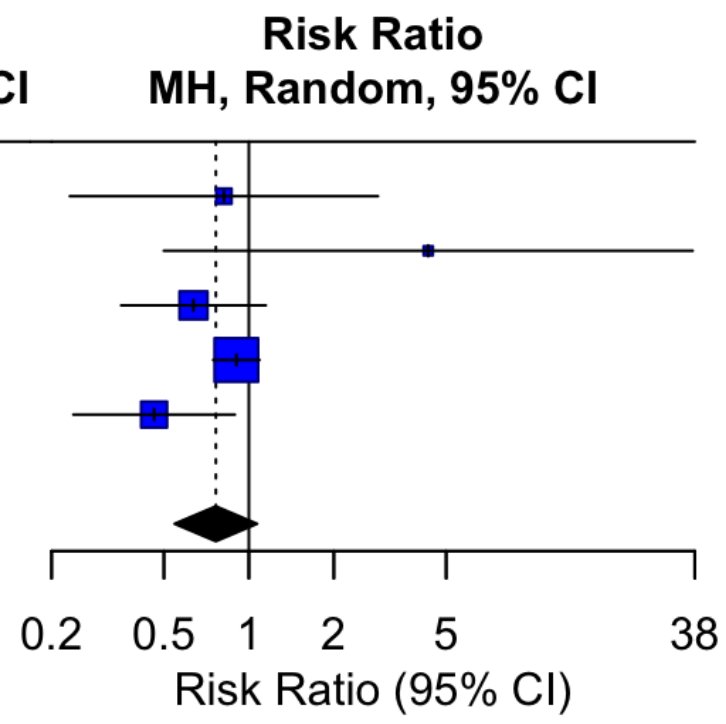

Figura 5S. Gráfico de floresta sobre mortalidade cardiovascular.
Desfecho avaliado através da análise combinada do número de eventos/n total de cada estudo.

Study	RR	Low 95% CI	High 95% CI	Pooled RR	Pooled 95% CI	Weight (%)
**Viés = Baixo**						
CAT	4.32	0.50	37.36	4.32	[0.50; 37.34]	1.2
DANISH	0.87	0.70	1.09	0.87	[0.70; 1.09]	49.1
**Random effects model**				**1.33**	**[0.33; 5.35]**	**50.2**
Heterogeneity: I^2 = 52.3%, τ^2 = 0.6722, p = 0.1474						
**Viés = Algumas considerações**						
AMIOVIRT	0.82	0.23	2.87	0.82	[0.23; 2.90]	3.3
DEFINITE Trial	0.46	0.24	0.89	0.46	[0.24; 0.89]	11.0
SCD-HeFT	0.68	0.44	1.03	0.68	[0.44; 1.04]	22.4
**Random effects model**				**0.62**	**[0.44; 0.87]**	**36.7**
Heterogeneity: I^2 = 0%, τ^2 = 0, p = 0.5580						
**Viés = Alto**						
COMPANION	0.64	0.35	1.15	0.64	[0.35; 1.16]	13.1
**Random effects model**				**0.75**	**[0.59; 0.95]**	**100.0**
Heterogeneity: I^2 = 25.1%, τ^2 = 0.0160, p = 0.2457						
Test for subgroup differences: χ^2_2 = 1.11, df = 2 (p = 0.5753)						

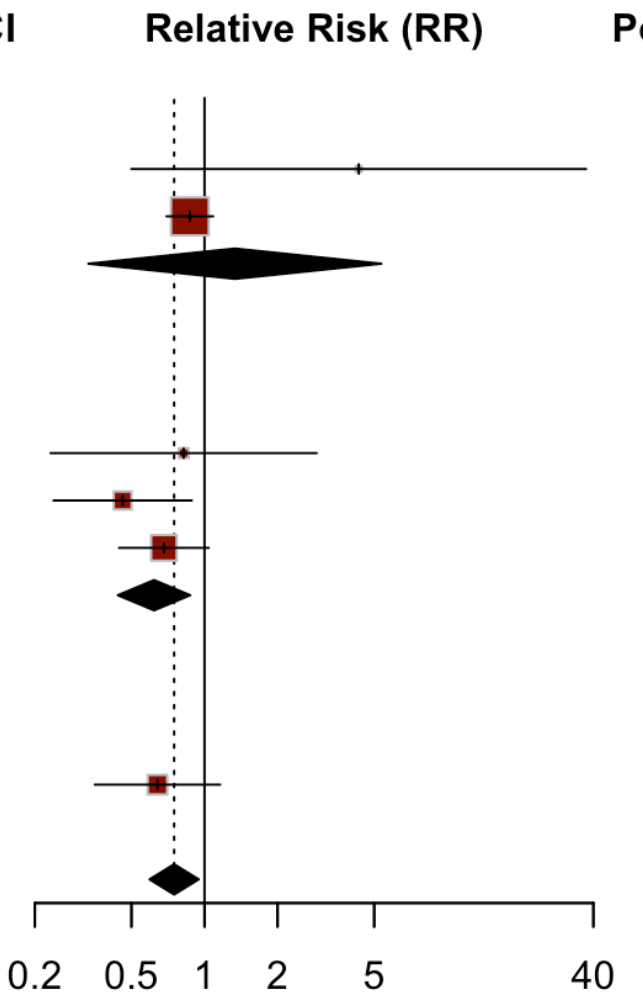

Figura 6S. Análise de subgrupo estratificada por avaliação do risco de viés para o desfecho mortalidade cardiovascular.

Desfecho avaliado através da análise combinada das medidas de efeito dos estudos incluídos.

Study	RR	Low CI95%	High CI95%
AMIOVIRT	0.87	0.32	2.42
CAT	0.83	0.45	1.52
COMPANION	0.51	0.30	0.89
DANISH	0.89	0.74	1.08
DEFINITE Trial	0.65	0.40	1.06
SCD-HeFT	0.97	0.79	1.20

Pooled RR = 0.84 [0.71; 1.00]

Heterogeneity: I^2 = 19.4%, τ^2 = 0.0095, p = 0.2872

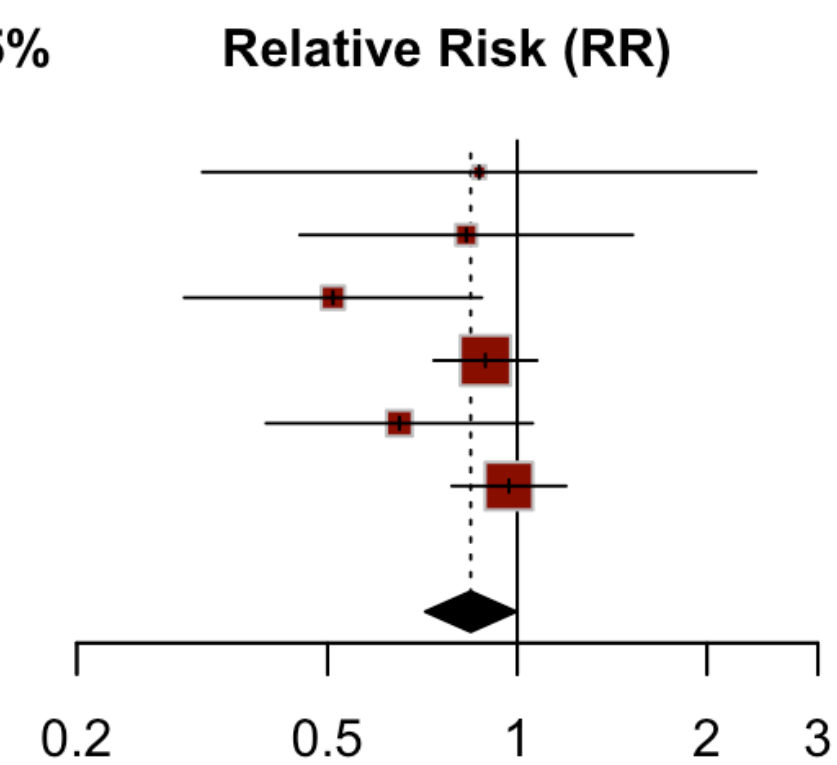

Figura 7S. Gráfico de floresta sobre mortalidade por todas as causas.

Desfecho avaliado através da análise combinada das medidas de efeito dos estudos incluídos (AMIOVIRT e CAT o cálculo do RR foi feito a partir do número de eventos/n total; COMPANION, DANISH, DEFINITE Trial e SCD-HeFT foi assumida a medida de efeito fornecida pelo estudo).

Study or Subgroup	Experimental Events	Experimental Total	Control Events	Control Total	Weight	Risk Ratio MH, Random, 95% CI
**Viés = Baixo**						
CAT	13	50	17	54	8.2%	0.83 [0.45; 1.52]
DANISH	208	556	226	560	32.6%	0.93 [0.80; 1.07]
**Total (95% CI)**		**606**		**614**	**40.8%**	**0.92 [0.80; 1.06]**
Heterogeneity: $Tau^2 = 0$; $Chi^2 = 0.13$, df = 1 (P = 0.7188); $I^2 = 0\%$						
**Viés = Algumas considerações**						
AMIOVIRT	6	51	7	52	3.4%	0.87 [0.32; 2.42]
DEFINITE Trial	28	229	40	229	13.0%	0.70 [0.45; 1.09]
SCD-HeFT	185	398	170	394	32.0%	1.08 [0.92; 1.26]
**Total (95% CI)**		**678**		**675**	**48.4%**	**0.92 [0.66; 1.29]**
Heterogeneity: $Tau^2 = 0.0427$; $Chi^2 = 3.29$, df = 2 (P = 0.1931); $I^2 = 39.2\%$						
**Viés = Alto**						
COMPANION	28	270	23	127	10.8%	0.57 [0.34; 0.95]
**Total (95% CI)**		**1554**		**1416**	**100.0%**	**0.88 [0.72; 1.07]**
Heterogeneity: $Tau^2 = 0.0249$; $Chi^2 = 8.45$, df = 5 (P = 0.1330); $I^2 = 40.8\%$						
Test for subgroup differences: $Chi^2 = 3.14$, df = 2 (P = 0.2079)						

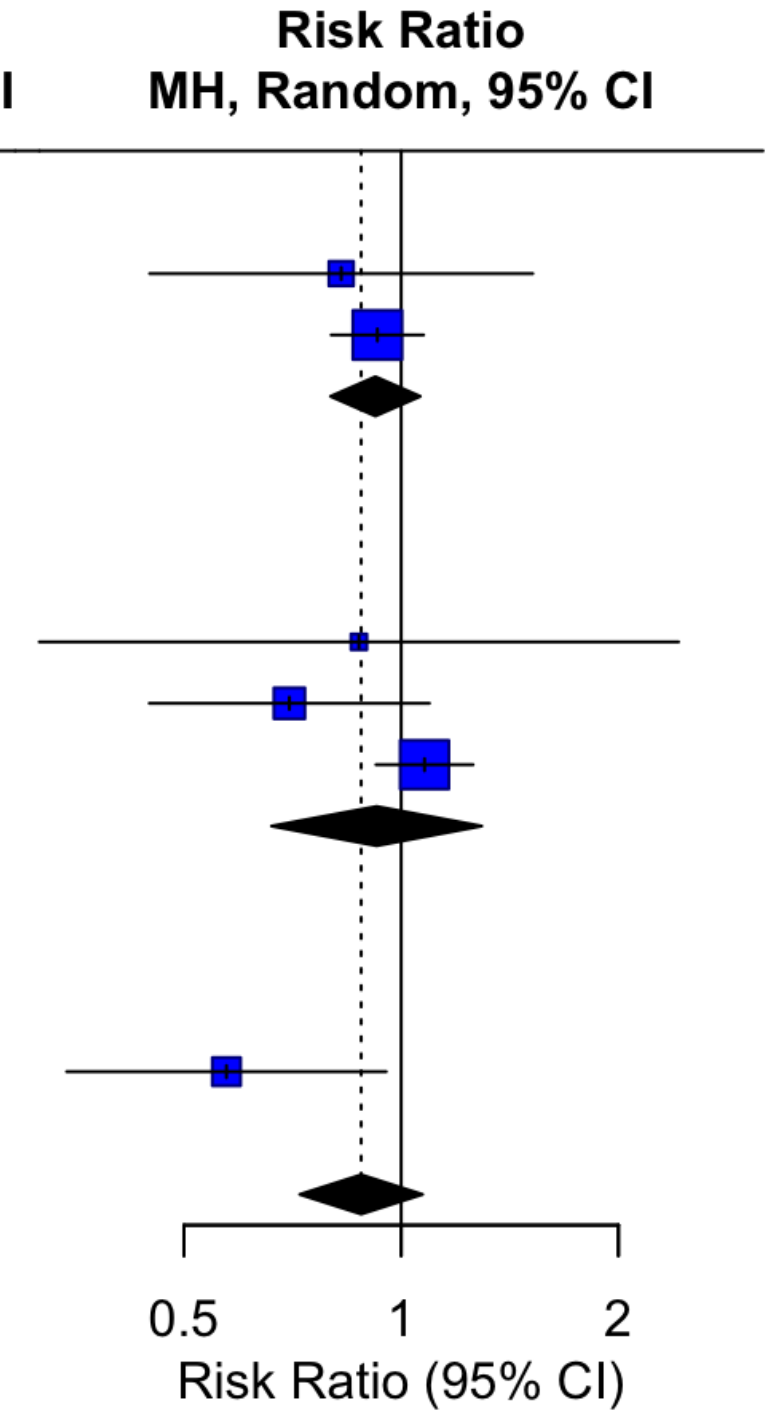

Figura 8S. Análise de subgrupo estratificada por avaliação do risco de viés para o desfecho mortalidade por todas as causas.

Study or Subgroup	Experimental Events	Experimental Total	Control Events	Control Total	Weight	Risk Ratio MH, Random, 95% CI
**Seguimento = < 6,8 anos**						
AMIOVIRT	6	51	7	52	3.4%	0.87 [0.32; 2.42]
CAT	13	50	17	54	8.2%	0.83 [0.45; 1.52]
COMPANION	28	270	23	127	10.8%	0.57 [0.34; 0.95]
DEFINITE Trial	28	229	40	229	13.0%	0.70 [0.45; 1.09]
**Total (95% CI)**		**600**		**462**	**35.3%**	**0.69 [0.52; 0.92]**
Heterogeneity: $Tau^2 = 0$; $Chi^2 = 1.06$, df = 3 (P = 0.7878); $I^2 = 0\%$						
**Seguimento = > 6,8 anos**						
DANISH	208	556	226	560	32.6%	0.93 [0.80; 1.07]
SCD-HeFT	185	398	170	394	32.0%	1.08 [0.92; 1.26]
**Total (95% CI)**		**954**		**954**	**64.7%**	**1.00 [0.86; 1.16]**
Heterogeneity: $Tau^2 = 0.0054$; $Chi^2 = 1.9$, df = 1 (P = 0.1680); $I^2 = 47.4\%$						
**Total (95% CI)**		**1554**		**1416**	**100.0%**	**0.88 [0.72; 1.07]**
Heterogeneity: $Tau^2 = 0.0249$; $Chi^2 = 8.45$, df = 5 (P = 0.1330); $I^2 = 40.8\%$						
Test for subgroup differences: $Chi^2 = 4.99$, df = 1 (P = 0.0255)						

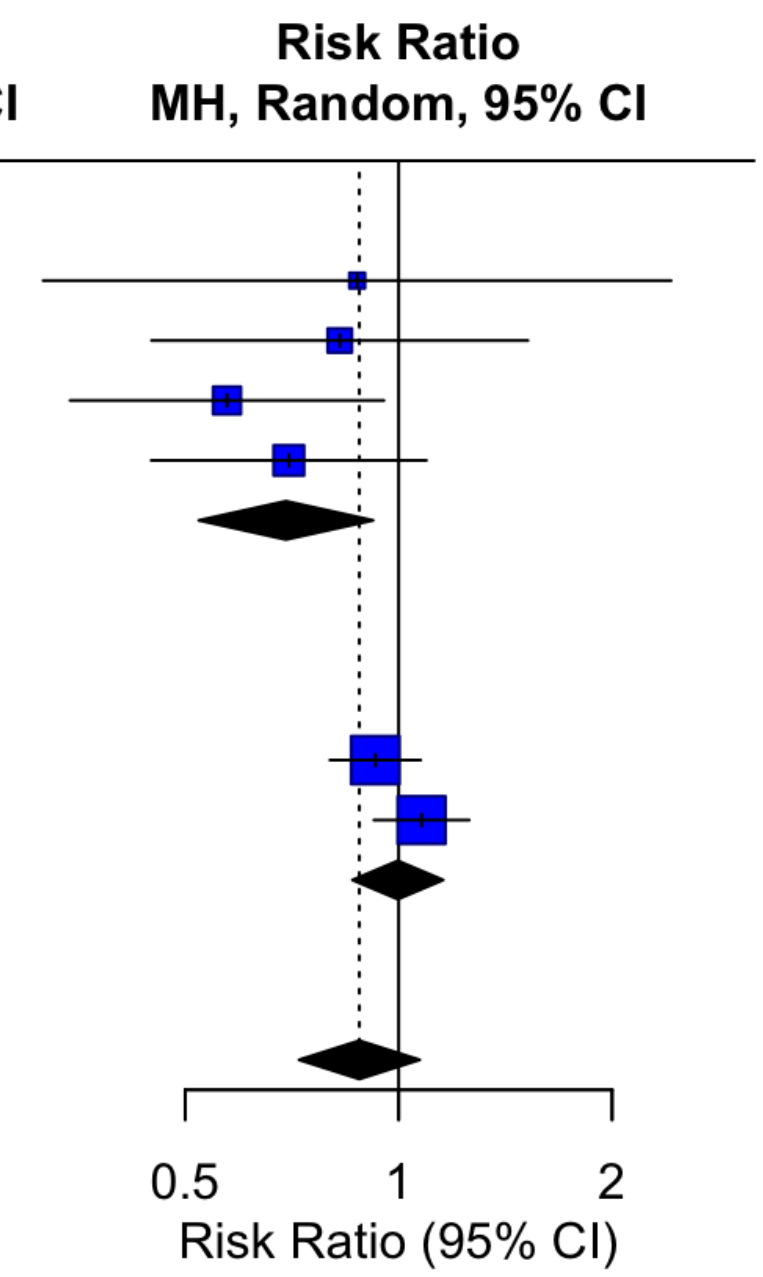

Figura 9S. Análise de subgrupo por média do tempo de seguimento para o desfecho mortalidade por todas as causas.

Gráfico de funil para desfecho mortalidade geral

Gráfico de funil para desfecho morte súbita

Gráfico de funil para desfecho morte cardiovascular

Figura 10S. Gráficos de Funil Ilustrativos.

(A)

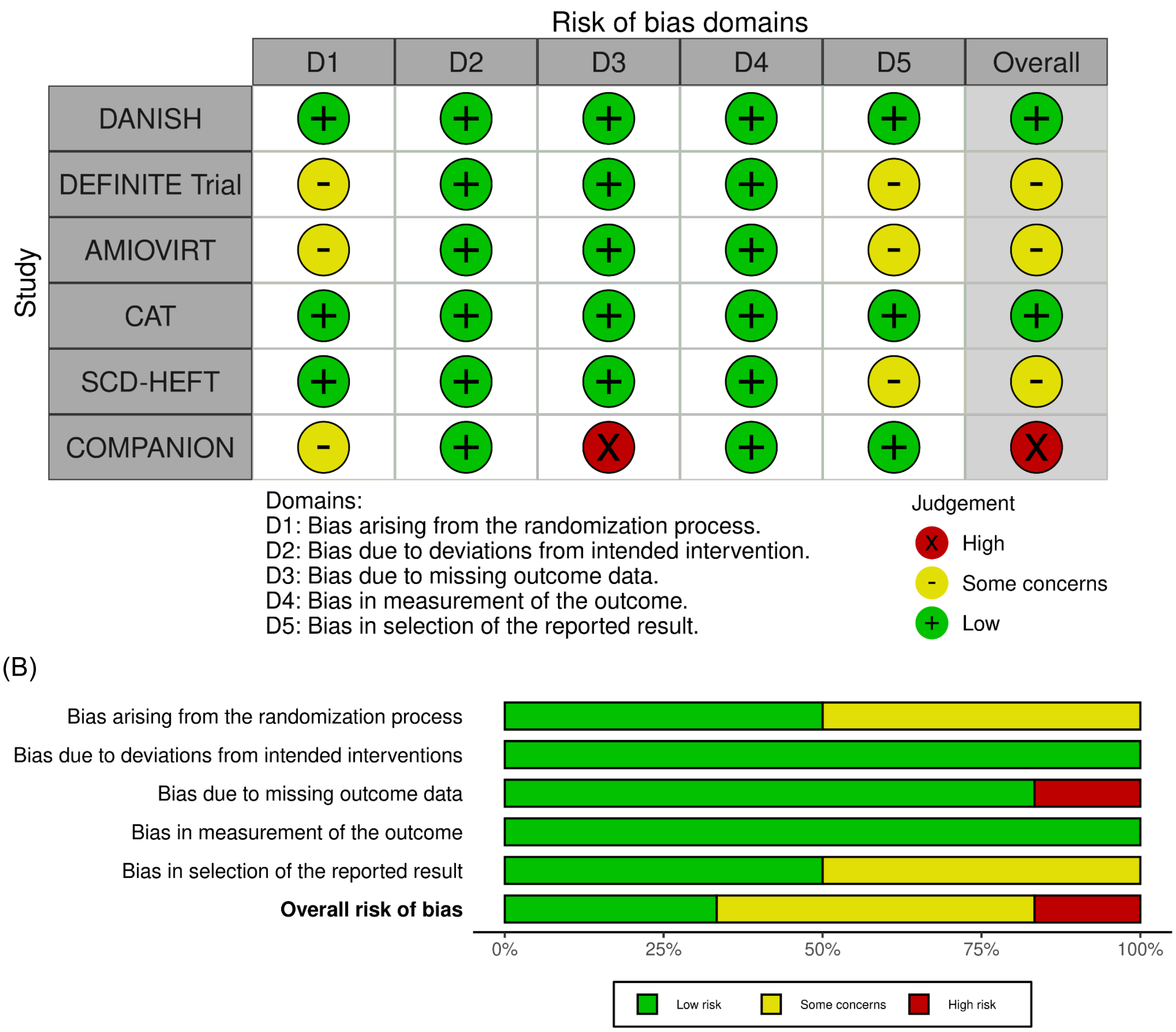

Figura 11S. Avaliação do risco de viés. (A) Avaliação por domínios da ferramenta Risk of Bias 2.0; (B) Sumário do risco de viés avaliado pela ferramenta Risk of Bias 2.0.

Legenda: A análise foi igual para todos os desfechos avaliados.
