# Supplementary material for: Eficácia de Cardiodesfibrilador Implantável na Prevenção Primária em Pacientes com Insuficiência Cardíaca com Fração de Ejeção Reduzida não Isquêmica
Source: Arq Bras Cardiol. 2026 Oct 1;123(7):e20250675. [Article in Portuguese] doi: 10.36660/abc.20250675 (PMC13668461; doi:10.36660/abc.20250675)
Supplement: Translation

# Translation: Effectiveness of Implantable Cardioverter-Defibrillator Therapy for Primary Prevention in Patients with Nonischemic Heart Failure with Reduced Ejection Fraction

**DOI:** 10.36660/abc.20250675i

**Authors:** Luis E. Rohde, Leandro I. Zimerman, Lídia Z. Moura, Luis C. Danzmann, Silvia M. Martins, Verônica Colpani, Kelly Zucatti, José Albuquerque de Figueiredo-Neto, Fabiana G. Marcondes-Braga, Ricardo Mourilhe-Rocha, Denilson Campos de Albuquerque, Mucio T. de Oliveira-Júnior, Marcus V. Simões

**Affiliations:** 1 Brazil Hospital de Clínicas de Porto Alegre, Porto Alegre, RS – Brazil; 2 Brazil Hospital Moinhos de Vento, Porto Alegre, RS – Brazil; 3 Brazil Hospital Universitário do Cajuru, Pontifícia Universidade Católica Paraná (PUCPR), Curitiba, PR – Brazil; 4 Brazil Santa Casa de Porto Alegre, Porto Alegre, RS - Brazil; 5 Brazil Universidade Luterana do Brasil, Canoas, RS – Brazil; 6 Brazil PROCAPE- Universidade de Pernambuco, Recife, PE – Brazil; 7 Brazil Hospital Sírio-Libanes, São Paulo, SP – Brazil; 8 Brazil Instituto de Cardiologia/ Fundação Universitária de Cardiologia, Porto Alegre, RS – Brazil; 9 Brazil Hospital Universitário da Universidade Federal do Maranhão, São Luiz, MA – Brazil; 10 Brazil Instituto do Coração (InCor), Hospital das Clínicas da Faculdade de Medicina da Universidade de São Paulo, São Paulo, SP – Brazil; 11 Brazil Universidade do Estado do Rio de Janeiro, Rio de Janeiro, RJ – Brazil; 12 Brazil Complexo Hospitalar Américas - Vitória e Samaritano Barra, Rio de Janeiro, RJ – Brazil; 13 Brazil Instituto D’Or de Pesquisa e Ensino-IDOR, Rio de Janeiro, RJ – Brazil; 14 Brazil Hospital das Clínicas da Faculdade de Medicina de Ribeirão Preto da Universidade de São Paulo, Ribeirão Preto, SP – Brazil

**Keywords:** Heart Failure, Implantable Defibrillators, Mortality

## Abstract

**Background:**

Primary prevention with an implantable cardioverter-defibrillator (ICD) has become an increasingly debated strategy, with its role in patients with nonischemic heart failure with reduced ejection fraction (HFrEF) undergoing continued reassessment.

**Objective:**

To summarize the available scientific evidence on the effectiveness of ICD therapy for primary prevention in patients with nonischemic HFrEF.

**Methods:**

A rapid systematic review and meta-analysis was conducted in accordance with the recommendations of the Cochrane Rapid Reviews Methods Group and the Preferred Reporting Items for Systematic Reviews and Meta-Analyses statement. Statistical significance was defined as a two-sided p-value < 0.05. Searches were performed in the MEDLINE, Embase, and CENTRAL databases. Randomized controlled trials (RCTs) evaluating ICD implantation for primary prevention were included, with sudden cardiac death (SCD), cardiovascular mortality, and all-cause mortality as the primary outcomes.

**Results:**

A total of 18 publications reporting daata from six pivotal RCTs were included. ICD therapy was associated with a statistically significant reduction in SCD (relative risk [RR] 0.41; 95%CI 0.26-0.65; high-certainty evidence) and cardiovascular mortality (RR 0.75; 95%CI 0.59-0.95; high-certainty evidence). For all-cause mortality, the primary analysis showed no statistically significant difference between groups (RR 0.88; 95%CI 0.72-1.07; moderate-certainty evidence). In the subgroup analysis, studies with shorter follow-up (< 6.8 years) demonstrated a significant reduction in all-cause mortality (RR 0.69; 95%CI 0.52-0.92).

**Conclusion:**

In patients with nonischemic HFrEF, ICD therapy reduced SCD and cardiovascular mortality but did not significantly reduce all-cause mortality in the primary analysis. These findings support the use of ICD therapy when guided by individualized clinical assessment.

## Introduction

Advances in the treatment of heart failure (HF) have led to a substantial decline in the absolute rates of sudden cardiac death (SCD) over the past 4 decades.^1^ Both pharmacological therapies^2,3^ and device-based interventions^4^ have been shown to reduce the risk of SCD and improve overall survival. Nevertheless, SCD continues to account for approximately 30%-40% of deaths reported in contemporary randomized controlled trials (RCTs) involving patients with HF with reduced ejection fraction (HFrEF).^5-7^

Implantable cardioverter-defibrillator (ICD) therapy is a cornerstone strategy for both the primary and secondary prevention of SCD, although its worldwide utilization remains substantially below guideline recommendations.^8^ However, the survival benefit of ICD therapy, particularly among patients with nonischemic cardiomyopathy, has been questioned following the publication of the DANISH trial,^9^ which demonstrated a reduction in the risk of SCD but no significant benefit with respect to all-cause mortality.

Since then, HF experts have debated the clinical scenarios in which ICD implantation should be recommended for patients with nonischemic cardiomyopathies, with the goal of identifying additional factors that may improve SCD risk stratification. Advanced age,^10^ the presence of myocardial fibrosis,^11^ and high-risk genotypes^12^ have emerged as important variables to support decision-making regarding ICD implantation, with the aim of optimizing the balance between clinical benefit, procedural risks, and healthcare costs.

In order to clarify the benefit of ICD implantation for the primary prevention of SCD in patients with nonischemic HFrEF based on the best available scientific evidence, the Department of Heart Failure of the Brazilian Society of Cardiology conducted the current meta-analysis.

## Methods

### Study design

This rapid systematic review was conducted in accordance with the recommendations of the Cochrane Rapid Reviews Methods Group^13^ and the Preferred Reporting Items for Systematic Reviews and Meta-Analyses statement.^14^ The review protocol was registered in the PROSPERO database.

### Outcomes of interest

The research question was formulated according to the PICO framework. The population (P) comprised patients with nonischemic HFrEF (left ventricular ejection fraction [LVEF] < 35%) without a history of malignant ventricular arrhythmias, other clinically significant structural heart diseases (eg, valvular heart disease), or previous episodes of aborted SCD, ventricular fibrillation, or hemodynamically unstable ventricular tachycardia (ie, secondary prevention). The intervention (I) was implantation of a transvenous or epicardial ICD, compared with no ICD implantation or standard medical therapy. Outcomes (O) included SCD, cardiovascular mortality, all-cause mortality, HF-related adverse events, and ICD-related complications, such as device pocket infection, infective endocarditis, and device explantation. Only RCTs were included because they represent the highest level of evidence for evaluating the efficacy of interventions while minimizing selection bias and confounding.

### Eligibility criteria

Inclusion criteria were i) RCTs enrolling patients with nonischemic HFrEF, LVEF < 35%, and an indication for primary prevention; ii) direct comparison between ICD therapy and standard medical therapy; and iii) reporting of at least one outcome of interest. Exclusion criteria were i) observational studies, narrative reviews, systematic reviews, and editorials; ii) studies including mixed populations without stratified analyses for the subgroup of interest; iii) studies evaluating exclusively other implantable devices, such as cardiac resynchronization therapy (CRT), conventional pacemakers, or ventricular assist devices; and iv) secondary analyses of the same RCTs that did not report new data.

### Literature search and study selection

Preliminary searches were conducted to identify systematic reviews addressing the topic of the present study in the MEDLINE, Embase, and CENTRAL databases. Detailed search methods and search strategies are provided in the Supplementary Material (Tables S1 and S2). Literature searches were performed on March 7, 2025, with no restrictions on language or year of publication.

Study selection was performed using Rayyan^15^ for duplicate removal and initial screening. The remaining records were subsequently assessed by one review author, with validation by a second review author. Full-text assessment and data extraction followed the same procedure.

A standardized data extraction form was developed to collect information on study identification, participant characteristics, inclusion and exclusion criteria, descriptions of the intervention and control groups, reported outcomes, duration of follow-up, funding sources, and potential conflicts of interest. When necessary, corresponding authors were contacted to obtain missing data.

### Methodological quality of the review

The systematic review selected for updating was identified based on the similarity of its review question to the clinical question addressed in the present evidence synthesis and on its methodological quality, as assessed using the AMSTAR 2 tool.^16^

### Risk of bias and certainty of the evidence

The risk of bias of the included RCTs was assessed using the Cochrane RoB 2 tool, which evaluates a predefined set of domains covering different aspects of the design, conduct, and reporting of RCTs. Domain-level judgments are generated through an algorithm based on responses to signaling questions and are classified as “low risk of bias,” “high risk of bias,” or “some concerns.”^17^

The certainty of the evidence was assessed using the GRADE approach,^18^ which considers study design limitations, risk of bias, inconsistency of results, imprecision of effect estimates, indirectness of evidence, and the likelihood of publication bias. The certainty of the evidence for each outcome was rated as i) high, ii) moderate, iii) low, or iv) very low.

### Statistical analysis

Data synthesis included both a narrative synthesis and meta-analysis for each outcome whenever sufficient data were available. Meta-analyses were performed using data from the included RCTs under a random-effects model in RStudio, version 2024.12.1+563. When necessary, missing data were imputed according to the recommendations of the Cochrane Handbook for Systematic Reviews of Interventions.^18^

For studies that did not report the mean follow-up duration but provided the median along with the first (Q1) and third (Q3) quartiles, the mean was estimated using the method proposed by Wan et al.^19^ The pooled mean follow-up across the included studies was subsequently calculated as a weighted average, using the sample size of each study as the weighting factor to appropriately reflect its relative contribution. For studies reporting only the median without additional information, such as quartiles or minimum and maximum values, the previously described estimation methods could not be applied. In these cases, the median was considered an approximation of the mean only when the sample size exceeded 70 participants.^19^

When the number of events was not reported, the approximate number of deaths in each treatment group was estimated from the cumulative event rates presented in the Kaplan-Meier curves.^20^ Relative risk (RR) was used as the summary measure of effect for pooled analyses. When only the effect estimate and its confidence interval were reported, the published values were used. To combine these estimates with studies reporting only the number of events and the total number of participants in each treatment arm, RR estimates were transformed to the natural logarithmic scale (log[RR]), and the corresponding standard errors were calculated for inclusion in the meta-analysis.

For studies reporting hazard ratio (HR), these estimates were incorporated into the pooled analyses as relative effect measures and analyzed on the natural logarithmic scale using inverse-variance methods. A 95%CI was adopted for all analyses.

Statistical heterogeneity was assessed using Cochran’s *Q* test, with p < .05 indicating statistically significant heterogeneity. Heterogeneity was further quantified using the Higgins I^2^ statistic, which estimates the proportion of total variability attributable to between-study heterogeneity. I^2^ values were interpreted according to the following thresholds: 0%-40%, heterogeneity might not be important; 30%-60%, moderate heterogeneity; 50%-90%, substantial heterogeneity; and 75%-100%, considerable heterogeneity.^18^

Publication bias was assessed using funnel plots and the Egger and Begg tests when a sufficient number of studies was available. Sensitivity analyses were also performed for the primary outcomes by excluding studies with at least one domain judged as having either a high risk of bias or some concerns.

## Results

### Study selection

The initial search, conducted to identify systematic reviews, retrieved 88 records across the databases. After removal of four duplicate records, 84 references underwent title and abstract screening. Of these, five articles were selected for full-text assessment, and three systematic reviews were ultimately included^21-23^ (Figure S1). Methodological quality was assessed using the AMSTAR 2 tool, which identified important methodological limitations in two of the reviews,^22,23^ whereas the third was rated as having high methodological quality^21^ (Table S3).

The search for RCTs identified 461 records. After removal of 41 duplicate records and exclusion of 347 studies during title and abstract screening, 73 articles were selected for full-text assessment. In addition, one record was identified through manual searching. At the end of the selection process, 18 publications were included in the systematic review, of which 12 represented follow-up reports from six pivotal RCTs. Of these, five publications were included in the meta-analysis of SCD,^24-28^ five in the meta-analysis of cardiovascular mortality,^24-26,28,29^ and six in the meta-analysis of all-cause mortality^24-26,28-30^ (Figure S2). More recent publications from the included studies that evaluated the outcomes of interest were also identified during the literature search. A complete list of excluded studies and the corresponding reasons for exclusion is provided in Table S4.

### Characteristics of the included studies

Data were extracted from six RCTs, the characteristics of which are summarized in Table 1. The studies were conducted between 1991 and 2020, and all employed a multicenter design involving sites in the United States,^24,28-32^ Germany,^29^ Denmark,^9^ Israel,^30^ Canada,^31^ and New Zealand.^33^ A total of 3,674 participants with nonischemic cardiomyopathy were included, with mean age ranging from 52 ± 11 years^27^ to 63 ± 12.6 years.^32^ Mean LVEF was comparable across studies, ranging from 21.4%^30^ to 25%.^9^ Of the six included RCTs, two compared ICD implantation with amiodarone or placebo,^24,32^ whereas the remaining trials used standard medical therapy or placebo as the control group.^9,28-30,32^ Follow-up duration ranged from 1 to 12 years.

**Table 1:** – Characteristics of the included RCTs

Characteristics	AMIOVIRT^24^	CAT^29^	COMPANION^32^	DANISH^9,26^	DEFINITE^27,30^	SCD-HeFT^4,28,31^
Study design	Open-label RCT	RCT	Open-label RCT	Open-label, parallel-group, controlled RCT	Investigator-initiated RCT based on observational data	Prospective primary prevention RCT
Setting	United States (10 centers)	Germany (15 centers)	United States (128 centers)	Denmark (five centers)	United States and Israel (48 centers)	Canada, New Zealand, and the United States (148 centers)
Study period and follow-up	Study period: August 1996 to June 2001. Mean follow-up: 24.0 ± 15.6 months.	Study period: July 1991 to March 1997. Mean follow-up: - All-cause mortality (5-year incidence): 66.0 ± 26.4 months. - Cardiovascular mortality (1-year incidence): 22.8 ± 4.3 months.	Study period: January 2000 to December 2002. Median follow-up: - All-cause mortality: 17.4 months in the CRT-P group, 16.9 months in the CRT-D group, and 16.0 months in the control group.	Study period: December 2007 to June 2016 (main trial). The extension study continued follow-up through May 2020. Median follow-up: 9.5 years (P25-P75, 7.9-10.9 years).	Study period: 2002 to 2004. Mean follow-up: 29.0 ± 14.4 months.	Study period: September 1997 to July 2001. Mean follow-up: - All-cause mortality: 11.0 ± 1.63 years. - SCD and cardiovascular mortality: 45.5 months.
Participants	n = 103 (70% male). Age: 59.0 ± 11.5 years. Clinical characteristics: Mean LVEF, 23%; mean duration of nonischemic dilated cardiomyopathy, 3.2 years; 83% of patients in NYHA class II or III.	n = 104 (78.9% male). Age: 52.0 ± 11.0 years. Clinical characteristics: Mean LVEF, 24%; 65.3% of patients in NYHA class II and 34.6% in NYHA class III; 96% receiving ACEi.	Total n = 1,520. Nonischemic subgroup: 682 (51.0% male). Age: 63.0 ± 12.6 years. Clinical characteristics: Median LVEF, 20%; 88% of patients in NYHA class III; median HF duration, 3.8 years; 41% with diabetes mellitus.	n = 1,116 (72.0% male). Age: 63.5 ± 12.2 years. Clinical characteristics: Mean LVEF, 25%; 53% of patients in NYHA class II, 45% in NYHA class III, and 1% in NYHA class IV; mean HF duration, 1.5 years; 19% with diabetes mellitus. Patients with a preexisting pacemaker or CRT device were also eligible provided they accepted a potential device upgrade.	n = 458 (71.2% male). Age: 58.3 ± 47.1 years*. Clinical characteristics: Mean LVEF, 21.4%; 21.6% of patients in NYHA functional class I, 57.4% in class II, and 21.0% in class III; mean HF duration, 2.83 years; 22.9% with diabetes mellitus.	Total n = 2,521. Nonischemic subgroup: 792 (77.0% male). Age: 60.0 ± 12.5 years. Clinical characteristics: Mean LVEF, 25%; 70% of patients in NYHA functional class II; 29% with diabetes mellitus, 17% with pulmonary disease, 52% with hypercholesterolemia, and 56% with hypertension.
Intervention	Amiodarone, initiated at 800 mg/day and reduced to 300 mg/day after 1 year. n = 52	ICD implantation. n = 50	(1) Optimized medical therapy + CRT-P. n = 617 n (nonischemic subgroup) = 285 (2) Optimized medical therapy + CRT-D. n = 595 n (nonischemic subgroup) = 270.	ICD implantation plus optimized medical therapy. n = 556	Single-chamber ICD implantation. n = 229	(1) Amiodarone: loading dose of 800 mg/day for 1 week, followed by 400 mg/day for 3 weeks, with subsequent adjustment of the maintenance dose according to body weight. n = 419 (2) Single-chamber ICD programmed in shock-only mode. n = 398
Comparator	ICD implantation. n = 51. Both groups received concomitant medical therapy with ACEi, beta-blockers, and potassium-sparing diuretics.	Standard medical therapy. n = 54. Both groups received concomitant therapy with ACEi and beta-blockers.	Optimized medical therapy consisting of ACEi, angiotensin receptor blockers, beta-blockers, and spironolactone, as tolerated, plus diuretics when clinically indicated. Digoxin and other HF medications were permitted at the investigator’s discretion. n = 308 n (nonischemic subgroup) = 127.	Usual medical therapy without ICD implantation, including an ACEi or angiotensin receptor blocker and a beta-blocker. n = 560	Standard medical therapy, including ACEi, beta-blockers, and other therapies as clinically indicated. n = 229	Placebo administered according to the same dosing schedule as amiodarone. n = 394 All groups received conventional medical therapy, including beta-blockers, ACEi, aldosterone antagonists, aspirin, and statins, when clinically indicated.
Outcomes^1^ and assessment schedule	Primary outcome: All-cause mortality. Secondary outcomes: SCD, nonsudden cardiac death, noncardiac death, syncope, arrhythmia-free survival, quality of life, and costs. Assessment schedule: Every 4 months.	Primary outcome: All-cause mortality. Secondary outcomes: Heart transplantation, cardiac mortality (classified as sudden or nonsudden), sustained ventricular tachycardia, and symptomatic ventricular tachyarrhythmias requiring antiarrhythmic therapy. Assessment schedule: Every 3 months.	Primary outcome: All-cause mortality or hospitalization. Secondary outcomes: All-cause mortality, cardiovascular mortality or hospitalization, HF-related mortality or hospitalization, device-related events, and clinical adverse events. Assessment schedule: From randomization until the first event.	Primary outcome: All-cause mortality. Secondary outcomes: SCD, cardiovascular death, resuscitated cardiac arrest, sustained ventricular tachycardia, changes in quality of life, and device-related infection. Assessment schedule: At 2 months and every 6 months thereafter.	Primary outcome: All-cause mortality. Secondary outcome: Sudden arrhythmic death. Assessment schedule: Every 3 months.	Primary outcome: All-cause mortality. Secondary outcomes: SCD, arrhythmic death, and hospitalization for congestive HF. Assessment schedule: Every 3 months.
Funding	Guidant	Guidant	Guidant	Medtronic and other funders	St. Jude Medical	National Heart, Lung, and Blood Institute; National Institutes of Health; Medtronic; Wyeth-Ayerst Laboratories; Knoll Pharmaceuticals.

1All studies used a two-sided significance level of 5% for the primary outcome. ACEi: angiotensin-converting enzyme inhibitor; CRT: cardiac resynchronization therapy; CRT-D: CRT with defibrillator; CRT-P: CRT with pacemaker; HF: heart failure; ICD: implantable cardioverter-defibrillator; LVEF: left ventricular ejection fraction; NYHA: New York Heart Association; RCT: randomized control*led trial; SCD: sudden cardiac death.*

### Sudden cardiac death

Four studies reported the raw number of events and evaluated the effect of ICD therapy on SCD in patients with HFrEF: AMIOVIRT, COMPANION, DANISH, and DEFINITE.^24-27^ Meta-analysis of these studies showed that, after a mean follow-up of 5.7 years, ICD therapy significantly reduced the risk of SCD compared with the control group (RR 0.44; 95%CI 0.24-0.80; high-certainty evidence; Figure S3).

The SCD-HeFT trial did not report the number of events for patients with nonischemic cardiomyopathy in its original publication but subsequently reported the corresponding effect estimate in a subgroup analysis.^28^ Inclusion of these data preserved the direction of the findings while increasing the precision of the pooled estimate, demonstrating a significant reduction in the risk of SCD (RR 0.41; 95%CI 0.26-0.65; high-certainty evidence) (Figure 1A).

Sensitivity analyses stratified according to the different effect measures reported in the original studies were also performed (Figure 1B; Figure 1C).

**Figure 1:** – Forest plots for the outcome of SCD. A) Primary analysis combining studies using the available effect estimates. For the AMIOVIRT and DEFINITE trials, RRs were calculated from the number of events and the total number of participants in each group, whereas for COMPANION, DANISH, and SCD-HeFT, the effect estimates reported by the study authors were used. B) Sensitivity analysis including only studies that reported RRs. C) Sensitivity analysis including only studies that reported HRs. df: degrees of freedom; HR: hazard ratio; IV: inverse variance; MH: Mantel-Haenszel; RR: relative risk; SCD: sudden cardiac death; SE: standard error.

Subgroup analysis evaluating the influence of risk of bias demonstrated a reduced RR for SCD across all strata. Although the magnitude of the effect varied, the benefit of ICD therapy remained consistent regardless of the risk-of-bias classification of the included studies (Figure S4).

### Cardiovascular mortality

Five RCTs reported cardiovascular mortality data for patients with nonischemic HFrEF.^24-26,28-30^ In the primary analysis, ICD therapy was not associated with a statistically significant reduction in the risk of cardiovascular death (RR 0.76; 95%CI 0.55-1.07), with moderate-certainty evidence because of imprecision.

The SCD-HeFT trial did not report the number of cardiovascular deaths stratified by ischemic and nonischemic etiology in its original publication but subsequently reported the corresponding effect estimate.^28^ Inclusion of these data resulted in a statistically significant reduction in the risk of cardiovascular death (RR 0.75; 95%CI 0.59-0.95). Figure 2 presents the results of both analyses.

**Figure 2:** – Forest plots for the outcome of cardiovascular mortality. A) Primary analysis combining studies using the available effect estimates. For the AMIOVIRT, CAT, COMPANION, and DEFINITE trials, RRs were calculated from the number of events and the total number of participants in each group, whereas for DANISH and SCD-HeFT, the effect estimates reported by the study authors were used. B) Sensitivity analysis including only studies that reported RRs. C) Sensitivity analysis including only studies that reported HRs. df: degrees of freedom; HR: hazard ratio; IV: inverse variance; MH: Mantel-Haenszel; RR: relative risk; SE: standard error.

Sensitivity analyses stratified according to the different effect measures reported in the original studies were also performed (Figure 2B; Figure 2C). A subgroup analysis evaluating the influence of risk of bias was also conducted, and the results are presented in Figure S6.

### All-cause mortality

Six RCTs evaluated the effect of ICD therapy on all-cause mortality in patients with nonischemic HFrEF: AMIOVIRT, CAT, COMPANION, DANISH, DEFINITE, and SCD-HeFT.^24-26,29-31^ The primary analysis showed no statistically significant benefit of ICD implantation, with evidence downgraded for imprecision. The absolute risk of all-cause mortality was 341 per 1,000 patients in the control group, corresponding to an absolute reduction of 41 deaths per 1,000 patients with ICD therapy (95%CI 24-96), with an RR of 0.88 (95%CI 0.72-1.07) (Figure 3).

**Figure 3:** – Forest plots for the outcome of all-cause mortality. A) Primary analysis combining studies using the number of events and the total number of participants in each group. B) Sensitivity analysis including only studies that reported RRs. C) Sensitivity analysis including only studies that reported HRs. df: degrees of freedom; HR: hazard ratio; IV: inverse variance; MH: Mantel-Haenszel; RR: relative risk; SE: standard error.

Sensitivity analyses stratified according to the different effect measures reported in the original studies were also performed (Figure 3B; Figure 3C). In a complementary analysis based on RR estimates, no statistically significant difference was observed (RR 0.84; 95%CI 0.71-1.00; I^2^ = 19.4%; p = 10.29) (Figure S7).

Exploratory analyses were conducted to better understand the influence of follow-up duration and concomitant CRT on the findings. When only the original SCD-HeFT publication^33^ was considered, replacing the longer-term follow-up report used in the primary analysis, ICD therapy was associated with a significant reduction in all-cause mortality compared with the control group (RR 0.81; 95%CI 0.69-0.95; I^2^ = 11.5%) (Figure 4). These findings suggest that inclusion of the extended follow-up report from SCD-HeFT substantially influences both the magnitude of the treatment effect and the heterogeneity of the meta-analysis.

**Figure 5:** – Forest plot for the outcome of all-cause mortality using data from the original DANISH and SCD-HeFT publications. df: degrees of freedom; HR: hazard ratio; IV: inverse variance; MH: Mantel-Haenszel; RR: relative risk; SE: standard error.

In an additional analysis using data from the original DANISH publication,^9^ which allowed inclusion of only patients who did not receive CRT, together with the original SCD-HeFT publication,^33^ ICD therapy was associated with a significant reduction in all-cause mortality compared with the control group (RR 0.78; 95%CI 0.66-0.91; I^2^ = 10%) (Figure 5).

**Figure 4:** – Forest plot for the outcome of all-cause mortality using data from the original SCD-HeFT publication. df: degrees of freedom; HR: hazard ratio; IV: inverse variance; MH: Mantel-Haenszel; RR: relative risk; SE: standard error.

A subgroup analysis evaluating the influence of risk of bias was also performed, and the results are presented in Figure S8.

Finally, subgroup analysis according to mean follow-up duration demonstrated a significant difference in the effect of ICD therapy on all-cause mortality. In studies with a mean follow-up shorter than 6.8 years,^24,25,29,30^ ICD therapy was associated with a statistically significant reduction in all-cause mortality, with no evidence of heterogeneity (RR 0.69; 95%CI 0.52-0.92; I^2^ = 10%). In contrast, no significant reduction in all-cause mortality was observed in studies with a mean follow-up longer than 6.8 years.^26,31^ The test for subgroup differences was statistically significant (p = 10.02), which suggests that follow-up duration may modify the effect of ICD therapy on this outcome. In addition, complications associated with long-term device use and reduced adherence to follow-up may have contributed to attenuating this benefit (Figure S9).

### Implantable cardioverter-defibrillator-related complications and heart failure-related events

Outcomes related to ICD complications and HF-related events were not pooled in meta-analyses because of limited data availability and substantial heterogeneity across studies. Therefore, these findings were synthesized narratively (Table S5).

ICD-related complications were reported across several studies, with device infection, inappropriate shocks, and mechanical complications being the most frequently described. Device infection was reported in two studies, with an incidence of 4.0% in the CAT trial and 4.9% in the DANISH trial.^9,29^ In the DANISH trial, major device infections, defined as those requiring lead extraction or posing a life-threatening risk, occurred in 2.7% of patients.^9^ Mechanical complications included cardiac perforation (2% in the CAT trial)^29^ and pneumothorax (2% in the DANISH trial).^9^

Rates of inappropriate shocks were reported in two studies, corresponding to 5.9% in DANISH^9^ and 21.4% in DEFINITE.^30^ Lead dislodgement or lead sensing/insulation failure occurred in 14% of patients in the CAT trial.^29^ In addition, the DEFINITE trial reported procedure-related complications, including one case each of hemothorax, pneumothorax, and cardiac tamponade^30^ (Table S5).

HF-related events were reported in three studies (AMIOVIRT, CAT, and DEFINITE).^24,29,30^ The incidence of syncope ranged from 3.9% to 17.0% in the ICD group and from 0% to 14.9% in the control group. Overall, syncope was a relatively common event in some studies but showed no consistent pattern of increased risk associated with ICD implantation (Table S6).

### Risk of bias and certainty of the evidence

The RoB 2 tool was applied to all outcomes of interest. Because all studies received the same risk-of-bias classification across the evaluated outcomes, the results are presented in a single consolidated figure (Figure S11).

Assessment of the randomization process indicated a low risk of bias for three studies (CAT, SCD-HeFT, and DANISH), whereas AMIOVIRT, DEFINITE, and COMPANION were judged as having some concerns. Despite the absence of blinding, no deviations from the intended interventions that could have affected the outcomes were identified, resulting in a low risk of bias for this domain. Only the COMPANION trial was judged to have a high risk of bias because of missing outcome data, whereas all remaining studies were classified as having a low risk of bias. Outcome measurement was judged to be at low risk of bias in all studies. Regarding selective reporting, three studies had prospectively published protocols that were consistent with the reported results, two did not report a prospectively registered protocol, and one study (DEFINITE) was judged as having some concerns regarding the selection of reported results.

Formal assessment of publication bias using the Egger and Begg tests was not feasible because fewer than 10 studies were included in each meta-analysis. Funnel plots were therefore generated for illustrative purposes only and are presented in Figure S10.

The certainty of the evidence was assessed using the GRADE approach (Table 2). Sudden cardiac death and cardiovascular mortality were classified as having high certainty of evidence. All-cause mortality was classified as having moderate certainty, indicating that the true effect is likely close to the estimate obtained, although there is a possibility of substantial differences. The reduction in certainty of evidence resulted from limitations related to the imprecision of the estimates.

**Table 2:** – Certainty of the evidence for ICD implantation for the primary prevention of mortality in patients with nonischemic heart failure with reduced ejection fraction

Outcome	Risk in the control group	Absolute effect with ICD (95%CI)	Relative effect (95%CI)	Participants (RCTs)	Certainty of the evidence (GRADE)	Interpretation
SCD Mean follow-up: 5.7 years	—	—	RR 0.41 (0.26-0.65)	Five RCTs	⨁⨁⨁⨁ High	ICD therapy reduces the risk of SCD.
Cardiovascular mortality Mean follow-up: 5.3 years	—	—	RR 0.75 (0.59-0.95)	Six RCTs	⨁⨁⨁⨁ High	ICD therapy reduces cardiovascular mortality.
All-cause mortality Mean follow-up: 6.8 years	341 per 1,000	41 fewer events per 1,000 (from 96 fewer to 24 more)	RR 0.88 (0.72-1.07)	In total, 2,970 participants (six RCTs)	⨁⨁⨁⊝^a^ Moderate	ICD therapy probably reduces all-cause mortality.

*The absolute effect was estimated from the observed risk in the control group and the relative effect of the intervention. ^a^The certainty of the evidence was downgraded by one level because of imprecision, as the 95%CI included both a clinically important benefit and no effect. ICD: implantable cardioverter-defibrillator; RCT: randomized controlled trial; RR: relative risk; SCD: sudden cardiac death.

## Discussion

In this systematic review and meta-analysis, ICD implantation in patients with nonischemic HFrEF was associated with a reduction in the risk of SCD, supported by high-certainty evidence, and a reduction in cardiovascular mortality, supported by moderate-certainty evidence. In contrast, the benefit with respect to all-cause mortality was less consistent and appeared to depend, at least in part, on the duration of follow-up. The analyses also identified implant-related complications, including device infection, pneumothorax, and inappropriate shocks as well as HF-related events such as syncope, with variable frequencies across studies. Collectively, these findings indicate that ICD implantation should not be recommended indiscriminately for all patients with nonischemic HFrEF.

Subgroup analyses demonstrated that the benefit of ICD therapy on all-cause mortality was more pronounced in studies with a follow-up duration of 6.8 years or less, whereas no statistically significant effect was observed in studies with longer follow-up. This cutoff was defined based on the mean follow-up duration of the studies included in the present review, which was longer than that adopted in the Cochrane review (36 months),^21^ which reflects the inclusion of more recent publications. These findings suggest that follow-up duration may influence the magnitude of the treatment effect, possibly because of disease progression and the increasing contribution of nonarrhythmic and noncardiovascular causes of death, which cannot be prevented by the device.

Another important consideration is the concomitant use of CRT in some of the included studies, particularly DANISH^9^ and COMPANION.^32^ In the COMPANION trial, all patients assigned to the intervention group received CRT combined with an ICD,^32^ whereas in the DANISH trial, approximately 58% of participants in both treatment groups received CRT.^9^ Because CRT reduces mortality and hospitalizations in patients with left ventricular systolic dysfunction and cardiac conduction abnormalities, its use may have attenuated the incremental benefit of ICD therapy. In COMPANION, the observed effects cannot be attributed exclusively to ICD therapy, thereby limiting interpretation of its independent contribution.^32^ Likewise, in DANISH, the widespread use of CRT may have contributed to the absence of a significant benefit of ICD therapy on all-cause mortality, as the effects of CRT may have reduced or overshadowed part of the expected benefit of defibrillator therapy.^9^ Furthermore, the effect of ICD therapy on all-cause mortality may be less apparent in contemporary clinical practice because of population aging and the widespread adoption of disease-modifying therapies, including angiotensin receptor-neprilysin inhibitors, sodium-glucose cotransporter 2 inhibitors, and CRT itself. Nevertheless, the complementary analysis performed in the present study demonstrated a reduction in mortality among both patients receiving CRT and those not receiving CRT.

Comparison of the findings of the present review with those of the systematic review by El Moheb et al.^21^ reveals substantial agreement regarding the effectiveness of ICD therapy. In that review, ICD implantation was associated with significant reductions in both all-cause mortality (HR 0.78; 95%CI 0.66-0.92) and SCD (HR 0.45; 95%CI 0.29-0.70). The present review incorporated more recent publications from previously analyzed RCTs and performed analyses stratified by outcome, risk of bias, and follow-up duration, allowing a more nuanced interpretation of the findings and the identification of potential effect modifiers. The smaller effect of ICD therapy on all-cause mortality observed in the present meta-analysis may be explained, at least in part, by the inclusion of studies with longer follow-up, in which the increasing competing risk of nonarrhythmic causes of death is likely to attenuate the relative effect of ICD therapy on overall mortality. Despite these differences, both reviews support the benefit of ICD therapy in preventing fatal arrhythmic events in patients with nonischemic HFrEF. Likewise, both suggest a potential benefit with respect to all-cause mortality, although this effect remains subject to statistical uncertainty and methodological differences among the included studies. Accordingly, caution is warranted when extrapolating this benefit to longer-term follow-up, reinforcing the need for individualized assessment of the risk-benefit profile when considering ICD implantation.

Therefore, considering additional risk stratifiers for SCD is essential in clinical practice. Although LVEF remains the most widely used criterion for ICD implantation, its predictive value is limited when used in isolation, particularly in patients with nonischemic HFrEF. Myocardial fibrosis, as assessed by late gadolinium enhancement on cardiac magnetic resonance imaging, has emerged as a robust marker of arrhythmic risk. A meta-analysis of 29 studies involving approximately 3,000 patients demonstrated a strong association between myocardial fibrosis and arrhythmic events.^33^ Furthermore, recent cohort studies have shown that myocardial fibrosis predicts arrhythmic risk more accurately than LVEF alone and that models combining fibrosis status with different LVEF thresholds outperform risk stratification based solely on LVEF.^11^ Other markers associated with an increased risk of SCD include a left ventricular end-diastolic diameter greater than 73 mm, the presence of exercise oscillatory ventilation during cardiopulmonary exercise testing, nonsustained ventricular tachycardia exceeding 10 beats on Holter monitoring, and elevated natriuretic peptide levels.^34^ More recently, genetic testing has also emerged as an important component of risk stratification. High-risk pathogenic variants, particularly in the *LMNA*, *PLN*, *FLNC*, *DSP*, and *RBM20* genes, have been associated with an increased risk of SCD, including in patients with an LVEF > 35%. Identification of these variants enables a more individualized approach to ICD implantation and facilitates cascade screening of at-risk family members.^12^ However, although these markers are recognized in international consensus statements, they have not yet been validated as inclusion criteria in RCTs evaluating ICD implantation in patients with HFrEF.

Another important consideration is the unequal access to ICD therapy and its cost-effectiveness, both in Brazil and internationally. Brazilian studies have reported relatively high incremental cost-effectiveness ratios for ICD implantation, although these estimates may become acceptable when the device is used following appropriate risk stratification.^35^

### Study limitations

This systematic review has several limitations that should be considered when interpreting its findings. First, although supported by methodological recommendations, the use of HRs as a proxy for RRs in some analyses may have introduced imprecision into the pooled estimates. In addition, clinical heterogeneity across studies, particularly with respect to follow-up duration, ICD implantation criteria, and baseline patient characteristics, may have influenced the results. The attenuation of the observed benefit in studies with follow-up longer than 6.8 years may reflect an increasing competing risk of death from other causes, aging of the study populations, and/or the greater use of contemporary disease-modifying therapies. This hypothesis should be regarded as exploratory and should not be interpreted as implying a causal relationship.

Meta-regression was not performed because of the limited number of included studies, which would have resulted in low statistical power and an increased likelihood of spurious associations. Although statistical heterogeneity was low to moderate in most analyses, unmeasured clinical differences, such as the use of different generations of ICDs across studies, may limit the generalizability of the findings. Finally, another important limitation is the limited availability of detailed data on ICD-related complications and HF-related events, which were reported descriptively in most studies, precluding quantitative synthesis.

## Conclusion

The findings of this systematic review support the benefit of ICD therapy for the primary prevention of SCD and cardiovascular mortality in patients with nonischemic HFrEF. However, no statistically significant reduction in all-cause mortality was observed. Longer follow-up duration, the competing risk of noncardiovascular death, concomitant use of CRT, and advances in contemporary pharmacological therapy appear to attenuate this benefit. Therefore, the findings do not support the routine use of ICD therapy in all symptomatic patients with nonischemic HFrEF receiving optimized guideline-directed medical therapy (Central Illustration). The decision to implant an ICD should be individualized, taking into account the patient’s clinical profile, concomitant therapies, and the risks inherent to the procedure. In addition, other risk stratification markers described in the literature may contribute to a more accurate identification of patients who are most likely to benefit from ICD therapy.

## Supplemental Materials

Supplemental Materials For additional information, please click here.
